# Predicting outcome in disorders of consciousness: A mega‐analysis

**DOI:** 10.1002/acn3.52061

**Published:** 2024-04-09

**Authors:** Yuri G. Pavlov, Franziska Spiegelsberger, Boris Kotchoubey

**Affiliations:** ^1^ Institute of Medical Psychology and Behavioral Neurobiology University of Tübingen Tübingen 72076 Germany

## Abstract

**Objective:**

Assessing recovery potential in patients with disorders of consciousness (DoC) is pivotal for guiding clinical and ethical decisions. We conducted a mega‐analysis of individual patient data to understand (1) if a time threshold exists, beyond which regaining consciousness is almost impossible, and (2) how recovery varies based on factors such as diagnosis, etiology, age, sex, and neuropsychological status.

**Methods:**

A systematic literature search revealed a total of 3290 patients. In this sample, we performed a Cox proportional hazards analysis for interval censored data.

**Results:**

We observed a late saturation of probability to regain consciousness in Kaplan–Meier curves, and the annual rate of recovery was remarkably stable, in that approximately 35% of patients regained consciousness per year. Patients in minimally conscious state (MCS) recovered more frequently than patients in unresponsive wakefulness syndrome (UWS). No significant difference was observed between the recovery dynamics of MCS subgroups: MCS+ and MCS−. Patients with hypoxic brain lesions showed worse recovery rate than patients with traumatic brain injury and patients with vascular brain lesions, while the latter two categories did not differ from each other. Male patients had moderately better chance to regain consciousness. While younger UWS patients recovered more frequently than older patients, it was not the case in MCS.

**Interpretation:**

Our findings highlight the necessity for neurologists to exercise caution when making negative predictions in individual cases, challenge traditional beliefs regarding recovery timelines, and underscore the importance of conducting detailed and prolonged assessments to better understand recovery prospects in DoC.

## Introduction

The prognosis for chronic severe disorders of consciousness (DoC), such as unresponsive wakefulness syndrome (UWS) and minimally conscious state (MCS), is an issue of high clinical and ethical significance. Given the absence of a definitive diagnostic gold standard (akin to biopsy in certain diseases), the diagnosis of DoC presents a well‐known challenge.^1^ Prognosis, therefore, serves as the primary reliable benchmark against which various diagnostic approaches are assessed. The question of prognosis is not only clinically relevant but also theoretically significant, providing insight into the brain's capacity to regenerate.

Traditionally, the prevailing assumption has been that no restoration of consciousness is viable beyond 1 year post‐ictum.^2^, ^3^ Nevertheless, recent studies challenge this notion, revealing instances of consciousness recovery in a UWS patient after 12 years^4^ and in an MCS patient after 19 years.^5^ Revising our beliefs on both short‐term and long‐term recovery outcomes is pivotal, as it informs critical adjustments in treatment strategies and decision‐making regarding the withdrawal of life‐sustaining measures. Furthermore, this shift in perspective has the potential not only to improve outcomes for patients and their families but also to reduce the financial burden associated with long‐term treatment.

Several key clinical predictors have been identified, with diagnosis emerging as the primary factor. Generally, patients diagnosed with MCS exhibit a more favorable prognosis for restoring consciousness compared to UWS patients.^6^, ^7^ Further stratification within the MCS category into MCS− and MCS+^8^ may offer additional predictive value,^9^, ^10^, ^11^ although this hypothesis is yet to undergo thorough testing. A more detailed description of the patients' neuropsychological status can be obtained through the Coma Recovery Scale‐Revised (CRS‐R)—a behavioral tool designed to assess auditory, visual, motor, oromotor/verbal, and communication functions and arousal level.^12^ CRS‐R subscales can potentially increase prognostic precision by considering the strongest predictors, instead of relying on a rough binary classification of the diagnosis. Moreover, certain subscales may prove more predictive than others. However, a common practice is to merge them into an overall score,^6^, ^13^, ^14^, ^15^ thus, limiting our understanding of the distinct predictive value of each subscale. Age and sex are readily available as potential predictors of recovery in DoC. Younger age has usually shown associations with better prognosis.^6^, ^16^, ^17^ Sex is rarely regarded as an important predictor, but it is possible that the null findings are due to limited sample size. The etiology of the disorder further differentiates recovery outcomes: traumatic brain injury (TBI) often leads to more favorable results,^6^, ^14^, ^18^ while anoxic brain injuries typically correspond to less favorable outcomes.^19^, ^20^ However, the significance of etiology in predicting outcomes was not universally confirmed in recent large‐scale studies.^13^, ^17^, ^21^

It is crucial to highlight that in individual studies and meta‐analyses, the primary emphasis is typically placed on the frequency of observed events, such as the recovery of consciousness. The time it takes for these events to occur is often overlooked. This oversight can lead to unintended consequences, where events may seem more frequent in patients with longer follow‐up times compared to those with shorter follow‐up times. To address this fundamental issue, we consolidated all available data from open sources and direct communication with authors to create an extensive dataset encompassing individual patient data and potential predictors. This dataset was then utilized in a mega‐analysis employing Cox regression models.

In this mega‐analysis (the analysis of raw data from multiple sources), we sought answers to the following questions: (i) What is the time threshold beyond which the probability of regaining consciousness becomes so low as to be practically close to zero? (ii) Does this trajectory of regaining consciousness differ based on diagnosis, etiology, age, sex, and neuropsychological status (CRS‐R subscales)?

## Methods

### Search strategy and selection criteria

#### Records

The search queries included a combination of (1) various names for the disorders of consciousness, excluding coma, and (2) terms for the outcome or prognosis. The exact search strings are reported in Supplementary Materials. Web of Science, PubMed, and SCOPUS databases were included in the search.

The initial search was executed on 17 January 2021. Following this search, we examined reference lists from papers published from 2011 onwards. This examination encompassed papers that had already been selected for data extraction at the time or were reviews pertaining to the topic of outcome prediction in DoC. This procedure (also known as “citation chasing”) was conducted on 28 August 2021. The only inclusion criterion was that the articles had to have DOI because of the “citationchaser” algorithm relied on them.^22^ To update the results, the second search was conducted on 28 January 2022.

Studies that explored factors for outcome prediction or pursued different objectives but reported outcome data at any point during follow‐up were considered eligible for inclusion. The exclusion criteria for the records were as follows:

1. descriptive (qualitative) articles, unless there was a potential for obtaining quantitative data from the authors;
2. review articles;
3. studies with direct interventions (e.g., transcranial brain stimulation, vagus nerve stimulation, but excluding medication);
4. coma as a diagnosis for all patients;
5. less than 14 days after injury for all patients; and
6. articles written in languages other than English, German, or Russian.

These criteria were applied during abstract screening (if the information was available at that stage) and during full‐text screening.

#### Patients

Data, typically presented in tabular form within the article or provided in Supplementary Materials, were extracted from the retained records following the application of exclusion criteria.

Subsequently, exclusion criteria specific to individual patients were implemented after incorporating the data into the summary spreadsheet. Patients were excluded from the analysis if:

1. the diagnosis was acute coma;
2. the time since onset was shorter than 14 days;
3. any of the following data were missing: time since onset, time interval between the diagnostic examination and the follow‐up examination, initial diagnosis, and the diagnosis at follow‐up;
4. patients died (if there was no indication of whether they improved before death);
5. patients were conscious at the initial assessment (including “emerged from minimally conscious state” (EMCS) diagnosis);
6. CRS‐R ratings were beyond possible ranges (e.g., 6 on the visual scale while the possible maximum is 5); and
7. there were discrepancies between the diagnosis provided and CRS‐R data (where available). For example, instances where CRS‐R indicated a patient as being in an EMCS while the stated diagnosis claimed they were in UWS.

### Data analysis

Below we describe how each key variable was handled in the analyses.

#### Time

The time since the onset of the injury that led to the loss of consciousness, and the duration of the follow‐up observation were included in each reported analysis.

For patients who had multiple observations, we retained all values (e.g., age and diagnosis) recorded during the first observation, with the exception of the follow‐up diagnosis (and/or CRS‐R) and the time since injury, for which we used the values from the last observation.

While some patients were followed up for longer than 10 years, the number of these patients was small (*n* = 39 or 1%). To contain predictions of our models within a maximum reasonable timeframe, we censored cases with longer follow‐up times but kept them in the models. Results of univariable analyses using all the available data without censoring, as well as models that used other time frames (e.g., 1 year), are available in Supplementary Materials.

#### Age and sex

When the age of a patient was presented as an interval (e.g., 30–40), which took place in <2% of cases, we used the mean of the range. When only the beginning or the end of the interval was indicated (e.g., >60), we treated the values as missing.

#### Etiology

The etiological groups were categorized into three main groups: vascular (including hemorrhage and stroke), anoxic, and TBI. Note that when all non‐TBI patients were included as a single group, the size of the group was larger than the sum of vascular and anoxic patients. The reasons were that, first, the non‐TBI group also included less common causes like encephalitis, infection, or intoxication; and second, that in some studies, only the classification of non‐TBI versus TBI was provided by the authors.

#### Diagnosis and Outcome

We categorized the initial diagnoses of all patients into one of four groups: UWS, MCS, MCS+, and MCS−. The latter two were rarely available and were used in one of the analyses. In cases where CRS‐R scales were accessible, we manually reclassified patients initially diagnosed as MCS into either MCS+ or MCS− categories. In the absolute majority of cases (80%) included in the analysis, the initial diagnosis was established using CRS‐R.

For the outcome diagnosis, we expanded the classification to include conscious states (which also included EMCS). The methodology for establishing the outcome diagnosis varied across the studies. Depending on the most reliable source of evidence available, we employed either the provided definition, the Glasgow Outcome Scale (GOS), or the CRS‐R, prioritizing CRS‐R when applicable. For GOS (or its extended version), a score of 3 or higher indicated a conscious state, a score of 2 signified no change in the patient's condition, and a score of 1 denoted death. The outcome diagnosis was primarily determined using CRS‐R (1473 cases, accounting for 44.8%). This was followed by the GOS(E) (542 cases, 16.5%) and modified versions of GOS, which included distinctions between MCS and UWS not present in the original GOS (178 cases, 5.4%). The diagnosis was defined without specifying the diagnostic tool in 580 cases (17.6%), and the Levels of Cognitive Functioning (LCF) scale was used in 345 cases (10.5%). Other diagnostic tools each accounted for less than 2% of the cases.

The resulting outcome variable was dichotomized, where a value of 1 denoted a conscious state (e.g., EMCS according to CRS or a score of 3 or higher on GOS), and a value of 0 was assigned in all other scenarios. MCS was never interpreted as a conscious state. As previously mentioned, any instances where the outcome was death were omitted from the primary analysis.

#### CRS index

For a more granular diagnosis definition, as an exploratory analysis, we used the CRS index^23^ to indirectly assess its predictive potential in comparison to the basic diagnostic approach. The CRS index ranges from 0 to 100, and we performed these calculations using the code provided by the authors.^24^ The index is designed in a way that the value of 8.315 would distinguish between UWS and MCS diagnosis. This particular value was solely utilized for stratifying the Kaplan–Meier curves.

### Statistics

We used semi‐parametric Cox proportional hazards models accounting for interval censored data for our main survival analyses. Standard errors were obtained using bootstrapping with 100 resamples. The R package *icenReg*,^25^ version 2.0.15 was used to fit the models.

*ir_clustBoot* function from *icenReg* package was used for a supplementary analysis taking into account the correlational structure of the data by clustering cases within individual records, rather than treating all observations as independent. We have presented the results of these cluster‐corrected models, both univariable and multivariable, in Supplementary Materials.

Most studies primarily assessed whether a patient regained consciousness by a predefined deadline rather than pinpointing the precise moment of recovery. Consequently, our dataset predominantly consists of interval‐censored data, signifying that we have information about whether patients improved or not within specific timeframes following their initial assessment. In instances where consciousness recovery occurred at a known time point, we applied right censoring to accommodate this. The rare cases where the exact day of improvement was known were treated as extremely narrow intervals, aligning with the overarching methodology.

## Results

### Records

**Figure 1:**
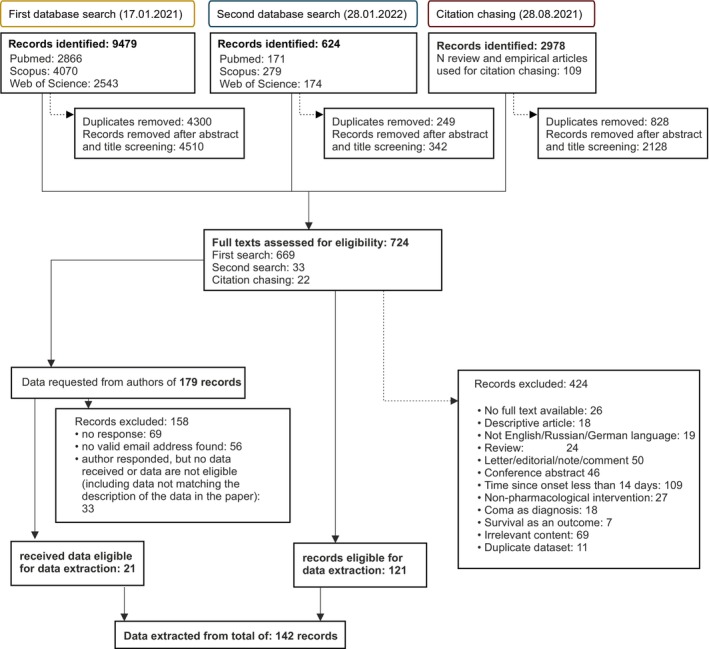
Flow chart of study selection.

Following the application of the aforementioned exclusion criteria, we identified 121 records that met the eligibility criteria for data extraction. Further 179 records were considered potentially eligible for data extraction; however, the required data were not available in either the article or its supplementary materials. To address this, we reached out to the authors of these articles with data requests. Among these requests, 21 authors provided the necessary data for extraction (please refer to Fig. 1 for a detailed breakdown). Consequently, we conducted our analysis on a total of 142 articles.

### Patients

Before exclusion, there were 4903 patients available for screening. After implementing our exclusion criteria, we had data from 3290 patients available for analysis, which we sourced from a total of 127 articles (see Supplementary Materials for the complete list). Of these 3290 patients, we received 1193 data points directly from authors via email.

### Descriptive statistics

Overall, the patients had a median age of 45 years, a median time since onset of 85 days, and median follow‐up time of 180 days (see Table 1 for subgroup statistics).

**Table 1:** Descriptive statistics.

	*n*	% regained consciousness	Estimated median time to event, days^a^	Median age, years	Median time since onset, days	Median follow‐up duration, days
Sex						
Male	2029	36.96	1440	44	90	180
Female	1056	32.10	1855	49	75	180
Diagnosis						
MCS	1312	58.38	484	45	90	180
MCS+	205	69.76	540	43	120	362
MCS−	672	53.87	540	46	90	180
UWS	1978	20.98	3735	46	81	180
Etiology						
TBI	1523	44.85	749	36	90	180
Non‐TBI	1766	28.14	2220	51	75	180
Anoxic	635	16.69	5042	46	75	185
Vascular	718	33.57	1108	55	75	180

^a^ Time after which 50% regained consciousness; obtained from unrestricted in time univariable Cox regression models.

About a third (36%; 1181/3290) of the patients eventually regained consciousness and this value remained stable over time. Given the median overall observation time of 1 year, the proportion of patients who regained consciousness within 1 year (37%; 676/1827) was very similar to the number between 1 and 5 years after injury (34%; 443/1304). Although the total number of patients was much smaller, this value remained comparable after 5 years (39%; 62/159). Stratifying the sample by diagnosis yielded similar consistency in both groups: (<1 year: UWS: 23%, MCS: 60%; 1–5 years: UWS: 18%, MCS: 56%; >5 years: UWS: 19%, MCS: 60%; see Table S1 for other subgroup stratification).

### Survival analysis

#### Diagnosis

**Figure 2:**
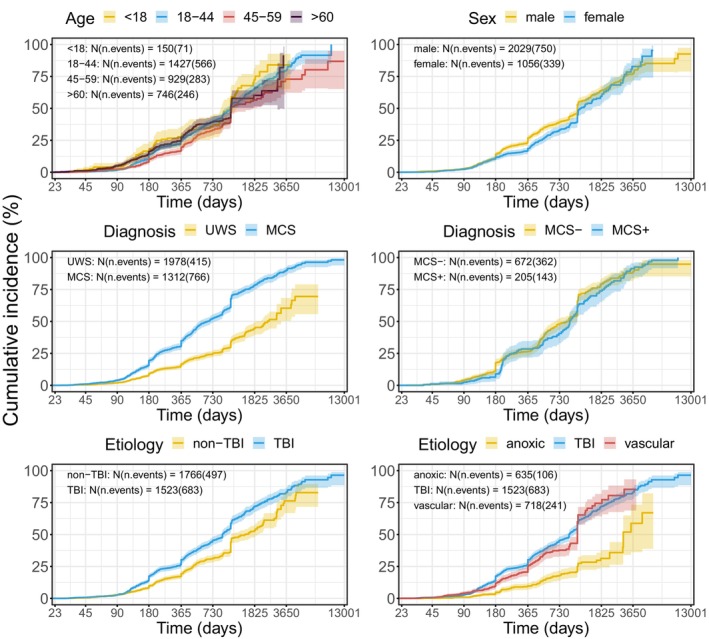
Kaplan–Meier curves illustrating the cumulative probability of regaining consciousness, stratified by age, sex, diagnosis, and etiology. To present the effect of age, data were grouped for illustrative purposes. In the analyses, however, they were taken as continuous.

Recovery chances of MCS patients were three times better as compared with UWS patients (Table 2 and Fig. 2).

**Table 2:** Cox regression results for age + sex + diagnosis (MCS/UWS) + etiology (TBI/non‐TBI) model.

Effect	*n*	HR (95% CI)
Age	3082	1 (1–1.01)
Sex		
Male	2028	
Female	1054	0.89 (0.77–1.02)
Diagnosis		
MCS	1260	
UWS	1822	0.33*** (0.29–0.38)
Etiology		
Non‐TBI	1667	
TBI	1415	1.37*** (1.21‐1.55)

HR, hazard ratio.

*** p < 0.001.

As an alternative to the binary diagnostic measure, the CRS index can be regarded as a continuous diagnostic variable. An analysis using this variable yielded similar results with a better CRS index being related to better outcome (see Tables S7 and S8).

The recovery course did not significantly differ between MCS+ and MCS− (Table 3 and Fig. 2).

**Table 3:** Cox regression results for age + sex + diagnosis (MCS+ vs MCS−) + etiology (TBI/non‐TBI) model.

Effect	*n*	HR (95% CI)
Age	842	1.01*** (1–1.01)
Sex		
Male	549	
Female	293	0.77* (0.63–0.95)
Diagnosis		
MCS−	657	
MCS+	185	1.06 (0.87–1.3)
Etiology		
Non‐TBI	419	
TBI	423	0.89 (0.73–1.09)

HR, hazard ratio.

* *p* < 0.05;

*** *p* < 0.001.

#### Etiology

Etiology was also a strong predictor. When considered as a binary category (TBI vs. non‐TBI), patients with TBI had a 37% better chance of recovery than non‐TBI patients (Table 2). When TBI, anoxic, and vascular etiologies were examined separately, both TBI and vascular (previously included in the non‐TBI category) increased the probability of recovery 2.2 times compared to anoxic patients (Table 4). However, no significant differences between TBI and vascular etiologies were found (HR (CI) = 1.02 (0.87–1.21) in the largest model with the diagnosis as the main predictor).

**Table 4:** Cox regression results for age + sex + diagnosis (MCS/UWS) + etiology (TBI/anoxic/vascular) model.

Effect	*n*	HR (95% CI)
Age	2693	1 (0.99–1)
Sex		
Male	1808	
Female	885	0.89 (0.76–1.03)
Diagnosis		
MCS	1084	
UWS	1609	0.35*** (0.3‐0.4)
Etiology		
Anoxic	596	
TBI	1415	2.27*** (1.81‐2.84)
Vascular	682	2.21*** (1.77‐2.76)

HR, hazard ratio.

*** *p* < 0.001.

#### Age

There was no consistent effect of age throughout the dataset. The formally obtained effect was unstable and its magnitude and significance varied depending on the model. Moreover, after correction for clustering, age effects completely disappeared (See Tables S9–S15 and S17).

An inspection of previous studies showing an age effect indicated that most of them included death as a negative outcome together with non‐recovery of consciousness. In contrast, we excluded patients who died at the end point because the cause of death remained unknown in most cases. To check the importance of this factor, we repeated the analysis adding the data of dead patients. As a result, age became a significant predictor of the outcome in some analyses (e.g., Age + Sex + Diagnosis + Etiology (2 levels) for 10 years follow‐up: *b* = −0.008, HR (CI) = 0.992 (0.989, 0.996), *p* < 0.001; Fig. S2), indicating that younger patients had better chances to regain consciousness. But this effect is confounded by the fact that older patients also died more frequently (death as the outcome; >45 years: 77% (513 patients), ≤45 years: 23% (154 patients)).

Next, we explored whether the effect of age was moderated by diagnosis, as previously suggested by Steppacher et al.^26^ Indeed, in UWS, a younger age was significantly and robustly associated with a positive outcome (univariable model after cluster correction: *b* = −0.016, HR (CI) = 0.984 (0.976, 0.993), *p* < 0.001), but this was not the case in MCS (*b* = 0.004, HR (CI) = 1.004 (0.995, 1.013), *p* = 0.422) (see also Table S18 for other models performed as robustness checks).

#### Sex

Summarizing evidence provided by univariable and multivariable models, male patients had significantly better chances of recovery than females. This effect of sex varied from 12% to 33% depending on other variables included in the model.

Combining the three important and reliable predictors described above (etiology, sex, and diagnosis), we found that, in absolute terms—not taking into account the time factor, MCS males with TBI were the most prospective group with 62% of them having eventually regained consciousness. The most negative prognosis was in UWS females with anoxic brain injury, with only 6% of them regaining consciousness.

#### CRS‐R scales

In the next analyses, we included CRS‐R scales and etiology with either two (*N* = 1558) or three levels (*N* = 1341) of the factor, while controlling for age and sex.

**Figure 3:**
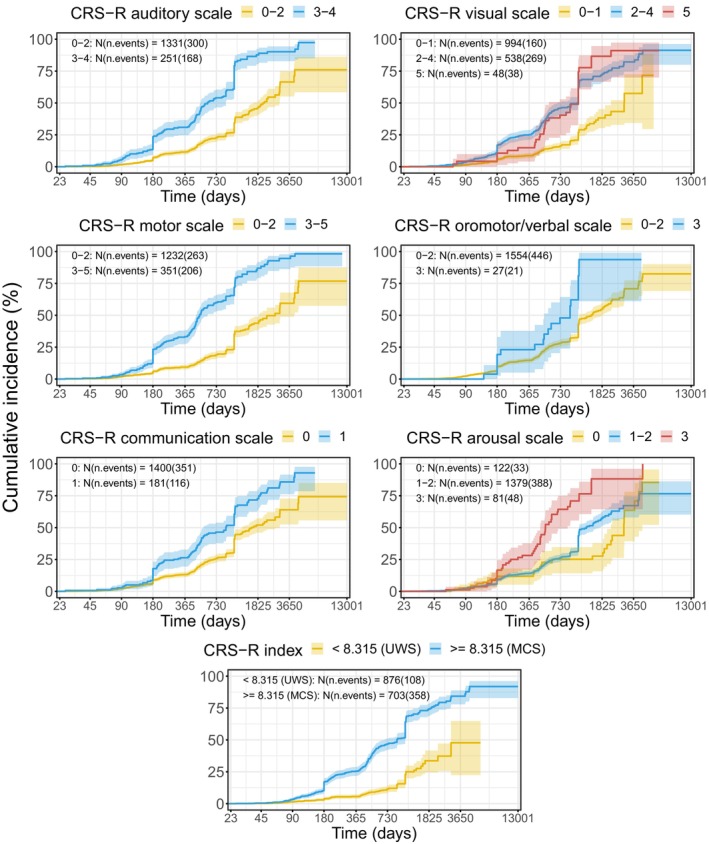
Kaplan–Meier curves illustrating the cumulative probability of regaining consciousness, stratified by CRS‐R scales and CRS‐R index. The values are grouped in a way to reflect changes in the diagnosis (e.g., UWS− > MCS or UWS− > MCS−) for all scales except arousal where there is no changing point and every value only classifies the score as UWS. In the case of arousal, middle values (eye‐opening with or without stimulation) are grouped. CRS‐R scores were grouped solely for illustrative purposes. In the analyses, they were taken as continuous.

Auditory, visual, and motor scales of CRS‐R consistently predicted a better outcome, especially long‐term (>1 year follow‐up observation; see Fig. 3, Tables 5 and 6). Each additional point on these scales increased the chances of recovery by 23–42%. The oromotor/verbal scale was more informative during the first year but lost its effect in the longer term (Tables S5 and S6). The arousal scale displayed a negative relation to the outcome, which can probably be explained by redistribution of variance among scales—in a univariate analysis the coefficient was positive. Communication scale had only two levels (Non‐functional: intentional vs. none) because the level “functional” would imply consciousness. The scale did not show any reliable relationship with the outcome.

**Table 5:** Cox regression results for age + sex + CRS scales + etiology (TBI/non‐TBI) model.

Effect	*n*	HR (95 CI)
Age	1558	1.01* (1–1.01)
Sex		
Male	973	
Female	585	0.75** (0.6–0.93)
CRS auditory	1558	1.27** (1.1–1.48)
CRS visual	1558	1.29*** (1.17–1.42)
CRS motor	1558	1.36*** (1.25–1.48)
CRS communication	1558	0.82 (0.62–1.08)
CRS oromotor/verbal	1558	1.07 (0.9–1.28)
CRS arousal	1558	0.8** (0.68–0.94)
Etiology		
Non‐TBI	951	
TBI	607	1.2 (0.96–1.49)

HR, hazard ratio.

* *p* < 0.05.

** *p* < 0.01

*** *p* < 0.001.

**Table 6:** Cox regression results for age + sex + CRS scales + etiology (TBI/anoxic/vascular) model.

Effect	*n*	HR (95 CI)
Age	1341	1 (1–1.01)
Sex		
Male	855	
Female	486	0.77* (0.63–0.95)
CRS auditory	1341	1.28** (1.09–1.51)
CRS visual	1341	1.23*** (1.12–1.35)
CRS motor	1341	1.42*** (1.28–1.58)
CRS communication	1341	0.85 (0.6–1.19)
CRS oromotor/verbal	1341	1.08 (0.91–1.3)
CRS arousal	1341	0.85 (0.72–1.01)
Etiology		
Anoxic	365	
TBI	607	2.02*** (1.43–2.85)
Vascular	369	2.37*** (1.63–3.44)

HR, hazard ratio.

* *p* < 0.05.

** *p* < 0.01.

*** *p* < 0.001.

## Discussion

First, we were looking for a “point of no return”, that is, the time point after which the recovery is highly improbable. Plateaus in Kaplan–Meier curves were reached very late, and the percentage of recovered patients remains surprisingly stable, regardless of the time elapsed since the injury (34–39% per year). It is important to keep in mind that survival analysis cannot pinpoint the exact moment when the probability of the target event reaches zero. Nevertheless, our results challenge a prevalent notion suggesting that recovery in DoC patients (particularly, UWS patients) is unlikely beyond the 1‐year mark.^3^, ^27^ However, our findings align with more recent extensive studies revealing a higher frequency of recovery 2 years post‐ictum than previously assumed.^21^, ^26^, ^28^, ^29^, ^30^ This has significant implications for research, highlighting the need to evaluate both established and emerging tools, including neuroimaging, for outcome prediction across extended follow‐up periods, spanning several years. From a practical standpoint, we can see how cautious a neurologist must be when making a negative prognosis for an individual patient.

Our study defined a favorable outcome as the patient's emergence from DoC. We are aware of the fact that this lump definition of “favorable” includes very different conditions from extremely severe cognitive and motor disabilities to fully independent life. At the present stage, however, predicting the level of independence or the severity of post‐recovery disabilities was not within the scope of our study. It is important to note that for patients and their relatives, merely transitioning to a conscious state might not equate to a satisfactory life. Therefore, while the instances of late recovery of consciousness might be more frequent than previously believed, future research should focus on providing more detailed predictions about the level of independence and the quality of life patients can expect post‐recovery.

Second, we examined whether neuropsychological and demographic variables affected the trajectory of regaining consciousness. Some recent large‐scale clinical trials and a meta‐analysis concluded that younger patients recover more frequently.^6^, ^17^, ^31^, ^32^ We were unable to confirm this finding in the overall sample. However, our additional analysis revealed that older patients die earlier and more frequently than younger patients. Yet, when alive, the former do not regain consciousness less frequently than the latter. A possible mechanism might be a self‐fulfilling prophecy,^33^, ^34^, ^35^ in which the treatment of older patients is frequently regarded as “medically futile,” leading to their faster death. Perhaps, the inclusion of death as a negative outcome is a key factor that could explain some previous findings regarding age and recovery. Another contributing factor is the diagnosis. In a subgroup analysis, we showed that younger age was predictive of a positive outcome in UWS patients only. This suggests that while age does influence the recovery trajectory in specific cases, such as UWS patients, overall age‐related differences in the probability of regaining consciousness may be more complex and influenced by external factors, including treatment decisions and attitudes toward older patients.

Sex of the patients emerged as a significant predictive factor for the outcome, though with a rather moderate effect size. While previous studies on the effect of sex have yielded inconsistent results,^16^, ^17^, ^26^ a recent large meta‐analysis aligns with our findings,^32^ suggesting that male patients, particularly husbands, may receive more effective care from their spouses than female patients. Although this factor can be real, it might result in a higher mortality rate among females rather than in an increased recovery rate in males. Biological factors, such as sex hormones, may play a role in the origin of the sex differences. While comprehensive evidence regarding the influence of sex hormones on outcomes in prolonged DoC remains elusive, there are notable findings in this area. For instance, estrogen and progesterone have been reported to exhibit neuroprotective effects at the time of TBI.^36^ This is further supported by findings that elevated serum estradiol levels are associated with improvement in CRS‐R scores.^37^ Another study highlighted the predictive role of testosterone levels in the recovery of consciousness post‐coma in male patients.^38^ More research on serum hormone levels is needed to fully understand their long‐term impact on the likelihood of regaining consciousness in prolonged DoC.

At first glance, our data appear to support the widely spread notion that TBI patients exhibit a more favorable prognosis than those with other etiologies (i.e., non‐TBI). A more detailed view reveals, however, that the apparent “TBI vs. non‐TBI” effect failed to account for the heterogeneity within the non‐TBI category. As we showed, patients with vascular brain lesions demonstrated recovery dynamics similar to TBI, challenging the generalized comparison. In contrast, anoxic patients exhibited the least favorable outcomes, worse than both TBI and vascular groups. These similarities between vascular and TBI‐caused DoC have already been noticed,^26^, ^39^ but in the present study, they are demonstrated in a large sample. Neuromorphological studies have shown substantial differences between the brain lesions underlying TBI and ischemic/anoxic DoC.^40^, ^41^, ^42^ Particularly, the former are related to a diffuse axonal injury, while the latter, to gray matter deficits in neocortex and hippocampus. However, a portion of vascular brain lesions also involve ischemic processes, but their consequences appear to be different. It is possible, therefore, that the decisive factor is not the exact nature of a lesion, but rather its size, in which hypoxic injuries are, generally, of a more global character than injuries related to other etiologies. This hypothesis concurs well with the idea of diminished or abnormal connectivity as a leading mechanism of DoC. Larger and more global lesions can be expected to disturb the connectivity pattern more than severe but focal damages. The vascular etiology is heterogeneous as well. Unfortunately, most original studies did not specify whether the vascular lesion was parenchymal or subarachnoidal, ischemic or hemorrhagic. Due to the wider spread of lesions associated with subarachnoidal hemorrhage, this etiology might be comparable to anoxic brain injury. These facts suggest the necessity of further, more detailed studies to comprehensively address the complexity within the non‐TBI category, particularly focusing on the diverse nature of vascular lesions.

As expected from earlier studies, MCS patients had substantially better trajectory of recovery of consciousness than UWS.^43^, ^44^, ^45^ Contrary to the expectation, however, there was no difference between the diagnoses MCS+ and MCS−. Given a sample of more than 800 patients, this zero effect can hardly be explained away by insufficient statistical power. Instead, it appears to represent a genuine phenomenon, suggesting that the distinction between MCS+ and MCS− may not carry implications for prognosis. Given the absence of a definitive gold standard in the domain of consciousness diagnostics, the prediction of patient outcomes emerges as the sole objective criterion available for evaluating diagnostic distinctions within the domain of DoC. The extent to which these distinctions possess a neurophysiological basis has been a subject of dispute for several years.^46^, ^47^, ^48^ This debate concerns the UWS/MCS dichotomy and, even more so, the finer MCS+/− distinction.^49^, ^50^, ^51^, ^52^ From this standpoint, our data clearly suggest that both UWS and MCS are “natural kinds,” regardless of whether they are viewed as dichotomous or continuous. Concerning the differentiation between MCS+ and MCS−, there are two possibilities: either the diagnostic distinctions between these conditions are spurious or they truly exist but have no bearing on the temporal progression of the disease.

CRS‐R was originally designed for diagnosing DoC, not for prognosis. Nevertheless, exploring how well its various subscales can forecast the recovery of consciousness is crucial. We found that three specific subscales—responses to sounds, visual stimuli, and motor activities—consistently exhibit correlations with favorable changes in recovery. Notably, our study indirectly supports the hypothesis that auditory localization is indicative of MCS.^53^ Indeed, in univariable models (Fig. 3; Table S19), we observed that a CRS‐R auditory scale score of 2 (“Localization to Sound”) predicts significantly higher chances of recovery than scores of 1 and 0, while a score of 1 does not demonstrate superior predictive value over a score of 0. A question should be discussed whether the auditory score 2 (and not 3, as in the present version) can be regarded as a sign of MCS. The interpretation of the remaining three subscales—communication, oromotor, and arousal—proved less straightforward. The limited variance in communication scale scores may explain the lack of its effect. The oromotor scale was unrelated to the long‐term outcome. Finally, the impact of the arousal scale becomes obscured by its covariations with other subscales. Understanding these nuances is essential for a comprehensive assessment of the CRS‐R's prognostic capabilities in different dimensions of recovery.

We acknowledge a potential limitation inherent in all studies involving data synthesis—the risk of systematic bias in the original studies. However, in the context of our study, the probability of such bias is deemed relatively low. This is primarily attributed to the fact that most original studies were centered around testing other hypotheses, with follow‐up data being only a by‐product of various research inquiries. As far as the importance of age, diagnosis, and etiology was not an object of the main hypotheses in a study, the data are not expected to be biased toward this hypothesis. Additionally, we recognized the importance of employing multiple analytical approaches to offer a comprehensive perspective. While the exploration of various models may lead to the emergence of spurious results, we are aware of this concern. To address it, we deliberately refrained from delving into effects that manifested themselves sporadically, focusing our discussion on those effects consistently observed across different analytic methodologies. This approach ensures that our interpretations prioritize the most robust and consistently replicated findings.

In conclusion, our findings refute the widely held belief that recovery beyond a year for DoC patients, especially those with UWS, is improbable, suggesting that recovery possibilities persist longer than previously assumed. The data suggest that TBI and vascular etiologies, as well as the MCS diagnosis, strongly improve the recovery trajectory. To enhance our insights into this domain, we recommend that future studies lengthen their follow‐up periods beyond the current median of 6 months, allowing for a more in‐depth examination of long‐term recovery trends in DoC patients.

## Conflict of Interest

None reported.

## Author Contributions

YGP: Conceptualization, Data curation, Validation, Investigation, Formal analysis, Project administration, Supervision, Visualization, Methodology, Writing—original draft, and Writing—review and editing. BK: Conceptualization, Methodology, Writing—original draft, and Writing—review and editing. FS: Data curation, Investigation.

## Supporting information


Data S1.
