# Supplementary material for: Predicting outcome in disorders of consciousness: A mega‐analysis
Source: Ann Clin Transl Neurol. 2024 Apr 9;11(6):1465–77. doi: 10.1002/acn3.52061 (PMC11187962; doi:10.1002/acn3.52061)
Supplement: Supplementary file 1 — Data S1. [file ACN3-11-1465-s001.pdf]

# Supplementary materials

## Search terms

**Pubmed**

("disorder of consciousness" OR "vegetative state" OR "unresponsive wakefulness" OR "minimally conscious" OR “post-coma unawareness” OR wachkoma OR "apalli* Syndrome” OR “apalli* syndrom*” OR “coma vigile” OR “post-coma unawareness” OR “postacute coma” OR “prolonged coma”) AND (predicti* OR recover* OR prognos* OR outcome OR follow-up)

***Scopus:***

TITLE-ABS-KEY ("disorder of consciousness"  OR  "vegetative state"  OR  "unresponsive wakefulness"  OR  "minimally conscious"  OR  "post-coma unawareness"  OR  wachkoma  OR  "apalli* Syndrome"  OR  "apalli* syndrom*"  OR  "coma vigile"  OR  "post-coma unawareness"  OR  "postacute coma"  OR  "prolonged coma" )  AND  TITLE-ABS-KEY ( predicti*  OR  recover*  OR  prognos*  OR  outcome  OR  follow-up )

***Web of Science:***

**TOPIC:** (("disorder of consciousness"  OR "vegetative state"  OR "unresponsive wakefulness" OR "minimally conscious"  OR “post-coma unawareness”  OR wachkoma  OR "apalli* Syndrome”  OR “apalli* syndrom*”  OR “coma vigile”  OR “post-coma unawareness”  OR “postacute coma”  OR “prolonged coma”)  AND (predicti*  OR recover*  OR prognos*  OR outcome  OR follow-up))

Timespan: All years. Indexes: SCI-EXPANDED, SSCI, A&HCI, CPCI-S, CPCI-SSH, BKCI-S, BKCI-SSH, ESCI, CCR-EXPANDED, IC.

## Included articles


1. Abe, H., Shimoji, K., Nagamine, Y., Fujiwara, S., & Izumi, S. I. (2017). Predictors of Recovery from Traumatic Brain Injury-Induced Prolonged Consciousness Disorder. Neural Plasticity, 2017, 9358092. https://doi.org/10.1155/2017/9358092
2. Amico, A. P., Terlizzi, A., Megna, M., Megna, G., & Damiani, S. (2013). Immune endocrinological evaluation in patients with severe vascular acquired brain injuries: Therapeutical approaches. Endocr Metab Immune Disord Drug Targets, 13(2), 204–208. https://doi.org/10.2174/18715303113130200009
3. Ammermann, Heidi, Jan Kassubek, Martin Lotze, Ernst Gut, Michael Kaps, Joachim Schmidt, Frank A. Rodden, und Wolfgang Grodd. „MRI Brain Lesion Patterns in Patients in Anoxia-Induced Vegetative State“. Journal of the

Neurological Sciences 260, Nr. 1–2 (September 2007): 65–70. https://doi.org/10.1016/j.jns.2007.03.026.

4. Arnaldi, D., Terzaghi, M., Cremascoli, R., De Carli, F., Maggioni, G., Pistarini, C., Nobili, F., Moglia, A., & Manni, R. (2016). The prognostic value of sleep patterns in disorders of consciousness in the sub-acute phase. Clin Neurophysiol, 127(2), 1445–1451. https://doi.org/10.1016/j.clinph.2015.10.042

5. Arts, W. F., van Dongen, H. R., & Meulstee, J. (1988). Unexpected improvement after prolonged post-traumatic vegetative state. Acta Neurochir Suppl (Wien), 44, 78–79. https://doi.org/10.1007/978-3-7091-9005-0_14

6. Arzi, A., Rozenkrantz, L., Gorodisky, L., Rozenkrantz, D., Holtzman, Y., Ravia, A., Bekinschtein, T. A., Galperin, T., Krimchansky, B. Z., Cohen, G., Oksamitni, A., Aidinoff, E., Sacher, Y., & Sobel, N. (2020). Olfactory sniffing signals consciousness in unresponsive patients with brain injuries. Nature, 581(7809), 428–433. https://doi.org/10.1038/s41586-020-2245-5

7. Ashwal, S., & Cranford, R. (2002). The minimally conscious state in children. Seminars in Pediatric Neurology, 9(1), 19–34. https://doi.org/10.1053/spen.2002.30333

8. Avesani, R., Gambini, M. G., & Albertini, G. (2006). The vegetative state: A report of two cases with a long-term follow-up. Brain Injury, 20(3), 333–338. https://doi.org/10.1080/02699050500487604

9. Babiloni, C., Sarà, M., Vecchio, F., Pistoia, F., Sebastiano, F., Onorati, P., Albertini, G., Pasqualetti, P., Cibelli, G., Buffo, P., & Rossini, P. M. (2009). Cortical sources of resting-state alpha rhythms are abnormal in persistent vegetative state patients. Clin Neurophysiol, 120(4), 719–729. https://doi.org/10.1016/j.clinph.2009.02.156

10. Bagnato, S., Boccagni, C., Prestandrea, C., Fingelkurts, A. A., Fingelkurts, A. A., & Galardi, G. (2017). Changes in Standard Electroencephalograms Parallel Consciousness Improvements in Patients With Unresponsive Wakefulness Syndrome. Archives of Physical Medicine and Rehabilitation, 98(4), 665–672. https://doi.org/10.1016/j.apmr.2016.09.132

11. Bagnato, S., Boccagni, C., Sant'angelo, A., & Galardi, G. (2013). A range of antiepileptic drugs do not affect the recovery of consciousness in vegetative and minimally conscious states. Epilepsy Behav, 27(2), 365–370. https://doi.org/10.1016/j.yebeh.2013.02.004

12. Bagnato, S., Boccagni, C., Sant'Angelo, A., Alito, A., & Galardi, G. (2018). Pain assessment with the revised nociception coma scale and outcomes of patients with unresponsive wakefulness syndrome: Results from a pilot study. Neurol Sci, 39(6), 1073–1077. https://doi.org/10.1007/s10072-018-3330-5

13. Bagnato, S., Boccagni, C., Sant'Angelo, A., Fingelkurts, A. A., Fingelkurts, A. A., & Galardi, G. (2017). Longitudinal Assessment of Clinical Signs of Recovery in Patients with Unresponsive Wakefulness Syndrome after Traumatic or Nontraumatic Brain Injury. Journal of Neurotrauma, 34(2), 535–539. https://doi.org/10.1089/neu.2016.4418

14. Bagnato, S., C. Boccagni, C. Prestandrea, A. Sant'Angelo, A. Castiglione, und G. Galardi. Prognostic value of standard EEG in traumatic and non-traumatic

disorders of consciousness following coma. Clinical Neurophysiology 121, Nr. 3 (März 2010): 274–80. https://doi.org/10/fqwfx2.

15. Bagnato, S., Minafra, L., Bravatà, V., Boccagni, C., Sant'angelo, A., Castiglione, A., Andriolo, M., Lucca, L. F., De Tanti, A., Pistarini, C., Formisano, R., Dolce, G., Gelfi, C., & Galardi, G. (2012). Brain-derived neurotrophic factor (Val66Met) polymorphism does not influence recovery from a post-traumatic vegetative state: A blinded retrospective multi-centric study. Journal of Neurotrauma, 29(11), 2050–2059. https://doi.org/10.1089/neu.2011.2183

16. Bagnato, S., Prestandrea, C., D'Agostino, T., Boccagni, C., & Rubino, F. (2021). Somatosensory evoked potential amplitudes correlate with long-term consciousness recovery in patients with unresponsive wakefulness syndrome. Clin Neurophysiol, 132(3), 793–799. https://doi.org/10.1016/j.clinph.2021.01.005

17. Bai, Y., Xia, X., Wang, Y., He, J., & Li, X. (2019). Electroencephalography quadratic phase self-coupling correlates with consciousness states and restoration in patients with disorders of consciousness. Clin Neurophysiol, 130(8), 1235–1242. https://doi.org/10.1016/j.clinph.2019.04.710

18. Bareham, Corinne A., Neil Roberts, Judith Allanson, Peter J.A. Hutchinson, John D. Pickard, David K. Menon, und Srivas Chennu. „Bedside EEG Predicts Longitudinal Behavioural Changes in Disorders of Consciousness". NeuroImage: Clinical 28 (2020): 102372. https://doi.org/10.1016/j.nicl.2020.102372.

19. Bekinschtein, T., Tiberti, C., Niklison, J., Tamashiro, M., Ron, M., Carpintiero, S., Villarreal, M., Forcato, C., Leiguarda, R., & Manes, F. (2005). Assessing level of consciousness and cognitive changes from vegetative state to full recovery. Neuropsychological Rehabilitation, 15(3–4), 307–322. https://doi.org/10.1080/09602010443000442

20. Billeri, L., Naro, A., Leo, A., Galletti, B., Tomasello, P., Manuli, A., Andronaco, V., Lauria, P., Bramanti, A., & Calabrò, R. S. (2019). Looking toward predicting functional recovery in disorders of consciousness: Can sensorimotor integration help us? Brain Injury, 33(3), 364–369. https://doi.org/10.1080/02699052.2018.1553309

21. Boccagni, C., Bagnato, S., Sant Angelo, A., Prestandrea, C., & Galardi, G. (2011). Usefulness of standard EEG in predicting the outcome of patients with disorders of consciousness after anoxic coma. J Clin Neurophysiol, 28(5), 489–492. https://doi.org/10.1097/WNP.0b013e318231c8c7

22. Boltzmann, M., Schmidt, S. B., Gutenbrunner, C., Krauss, J. K., Höglinger, G. U., & Rollnik, J. D. (2022). One-year outcome of brain injured patients undergoing early neurological rehabilitation: A prospective observational study. BMC Neurol, 22(1), 30. https://doi.org/10.1186/s12883-022-02549-w

23. Boltzmann, M., Schmidt, S. B., Gutenbrunner, C., Krauss, J. K., Stangel, M., Höglinger, G. U., Wallesch, C. W., & Rollnik, J. D. (2021). The influence of the CRS-R score on functional outcome in patients with severe brain injury receiving early rehabilitation. BMC Neurol, 21(1), 44. https://doi.org/10.1186/s12883-021-02063-4

24. Bruno, M. A., Vanhaudenhuyse, A., Schnakers, C., Boly, M., Gosseries, O., Demertzi, A., Majerus, S., Moonen, G., Hustinx, R., & Laureys, S. (2010). Visual fixation in the

vegetative state: An observational case series PET study. BMC Neurology, 10, 35. https://doi.org/10.1186/1471-2377-10-34

25. Carriere, M., Cassol, H., Aubinet, C., Panda, R., Thibaut, A., Larroque, S. K., Simon, J., Martial, C., Bahri, M. A., Chatelle, C., Martens, G., Chennu, S., Laureys, S., & Gosseries, O. (2020). Auditory localization should be considered as a sign of minimally conscious state based on multimodal findings. Brain Commun, 2(2). https://doi.org/10.1093/braincomms/fcaa194

26. Castro, M., Tillmann, B., Luauté, J., Corneyllie, A., Dailler, F., André-Obadia, N., & Perrin, F. (2015). Boosting Cognition With Music in Patients With Disorders of Consciousness. Neurorehabilitation and Neural Repair, 29(8), 734–742. https://doi.org/10.1177/1545968314565464

27. Chennu, S., Annen, J., Wannez, S., Thibaut, A., Chatelle, C., Cassol, H., Martens, G., Schnakers, C., Gosseries, O., Menon, D., & Laureys, S. (2017). Brain networks predict metabolism, diagnosis and prognosis at the bedside in disorders of consciousness. Brain, 140(8), 2120–2132. https://doi.org/10.1093/brain/awx163

28. Chillura, A., Naro, A., Micchia, K., Bramanti, A., Bramanti, P., & Calabrò, R. S. (2017). The value of midbrain morphology in predicting prognosis in chronic disorders of consciousness: A preliminary ultrasound study. Journal of the Neurological Sciences, 380, 46–50. https://doi.org/10.1016/j.jns.2017.07.002

29. Coleman, M. R., Davis, M. H., Rodd, J. M., Robson, T., Ali, A., Owen, A. M., & Pickard, J. D. (2009). Towards the routine use of brain imaging to aid the clinical diagnosis of disorders of consciousness. Brain, 132(Pt 9), 2541–2552. https://doi.org/10.1093/brain/awp182

30. Cui, Y., Song, M., Lipnicki, D. M., Yang, Y., Ye, C., Fan, L., Sui, J., Jiang, T., & He, J. (2018). Subdivisions of the posteromedial cortex in disorders of consciousness. Neuroimage Clin, 20, 260–266. https://doi.org/10.1016/j.nicl.2018.07.025

31. da Conceição Teixeira, L., Gill-Thwaites, H., Reynolds, F., & Duport, S. (2018). Can behavioural observations made during the SMART assessment detect the potential for later emergence from vegetative state? Neuropsychological Rehabilitation, 28(8), 1340–1349. https://doi.org/10.1080/09602011.2016.1243482

32. De Tanti, A., Saviola, D., Basagni, B., Cavatorta, S., Chiari, M., Casalino, S., De Bernardi, D., & Galvani, R. (2016). Recovery of consciousness after 7 years in vegetative state of non-traumatic origin: A single case study. Brain Injury, 30(8), 1029–1034. https://doi.org/10.3109/02699052.2016.1147078

33. Dewar, B. K., Pickard, J. D., & Wilson, B. A. (2008). Behavioural and psychosocial outcome following vegetative and minimally conscious states: Long-term follow-up. Brain Impairment, 9(3), 267–273. https://doi.org/10.1375/brim.9.3.266

34. Dhamapurkar, S. K., Rose, A., Florschutz, G., & Wilson, B. A. (2016). The natural history of continuing improvement in an individual after a long period of impaired consciousness: The story of I.J. Brain Injury, 30(2), 230–236. https://doi.org/10.3109/02699052.2015.1094131

35. Di, H. B., Yu, S. M., Weng, X. C., Laureys, S., Yu, D., Li, J. Q., Qin, P. M., Zhu, Y. H., Zhang, S. Z., & Chen, Y. Z. (2007). Cerebral response to patient's own name in the

vegetative and minimally conscious states. Neurology, 68(12), 895–899. https://doi.org/10.1212/01.wnl.0000258544.79024.d0

36. Edlow, B. L., Giacino, J. T., Hirschberg, R. E., Gerrard, J., Wu, O., & Hochberg, L. R. (2013). Unexpected recovery of function after severe traumatic brain injury: The limits of early neuroimaging-based outcome prediction. Neurocritical Care, 19(3), 364–375. https://doi.org/10.1007/s12028-013-9870-x

37. Eilander, H. J., Wijnen, V. J., Schouten, E. J., & Lavrijsen, J. C. (2016). Ten-to-twelve years after specialized neurorehabilitation of young patients with severe disorders of consciousness: A follow-up study. Brain Injury, 30(11), 1302–1310. https://doi.org/10.3109/02699052.2016.1170881

38. Estraneo, A., De Bellis, F., Masotta, O., Loreto, V., Fiorenza, S., Lo Sapio, M., & Trojano, L. (2019). Demographical and clinical indices for long-term evolution of patients in vegetative or in minimally conscious state. Brain Injury, 33(13–14), 1633–1639. https://doi.org/10.1080/02699052.2019.1658220

39. Estraneo, A., Fiorenza, S., Magliacano, A., Formisano, R., Mattia, D., Grippo, A., Romoli, A. M., Angelakis, E., Cassol, H., Thibaut, A., Gosseries, O., Lamberti, G., Noé, E., Bagnato, S., Edlow, B. L., Chatelle, C., Lejeune, N., Veeramuthu, V., Bartolo, M., … Trojano, L. (2020). Multicenter prospective study on predictors of short-term outcome in disorders of consciousness. Neurology, 95(11), e1488–e1499. https://doi.org/10.1212/wnl.0000000000010254

40. Estraneo, A., Loreto, V., Masotta, O., Pascarella, A., & Trojano, L. (2018). Do Medical Complications Impact Long-Term Outcomes in Prolonged Disorders of Consciousness? Archives of Physical Medicine and Rehabilitation, 99(12), 2523-2531.e3. https://doi.org/10.1016/j.apmr.2018.04.024

41. Estraneo, A., Moretta, P., Loreto, V., Santoro, L., & Trojano, L. (2014). Clinical and neuropsychological long-term outcomes after late recovery of responsiveness: A case series. Archives of Physical Medicine and Rehabilitation, 95(4), 711–716. https://doi.org/10.1016/j.apmr.2013.11.004

42. Falk, R. H. (1990). Physical and intellectual recovery following prolonged hypoxic coma. Postgrad Med J, 66(775), 384–386. https://doi.org/10.1136/pgmj.66.775.383

43. Faran, S., Vatine, J. J., Lazary, A., Ohry, A., Birbaumer, N., & Kotchoubey, B. (2006). Late recovery from permanent traumatic vegetative state heralded by event-related potentials. J Neurol Neurosurg Psychiatry, 77(8), 998–1000. https://doi.org/10.1136/jnnp.2005.076553

44. Faugeras, F., Rohaut, B., Valente, M., Sitt, J., Demeret, S., Bolgert, F., Weiss, N., Grinea, A., Marois, C., Quirins, M., Demertzi, A., Raimondo, F., Galanaud, D., Habert, M. O., Engemann, D., Puybasset, L., & Naccache, L. (2018). Survival and consciousness recovery are better in the minimally conscious state than in the vegetative state. Brain Injury, 32(1), 72–77. https://doi.org/10.1080/02699052.2017.1364421

45. Fernández-Espejo, D., Junque, C., Cruse, D., Bernabeu, M., Roig-Rovira, T., Fábregas, N., Rivas, E., & Mercader, J. M. (2010). Combination of diffusion tensor and functional magnetic resonance imaging during recovery from the vegetative state. BMC Neurology, 10, 77. https://doi.org/10.1186/1471-2377-10-76

46. Fingelkurts, A. A., Fingelkurts, A. A., Bagnato, S., Boccagni, C., & Galardi, G. (2016). Long-Term (Six Years) Clinical Outcome Discrimination of Patients in the Vegetative State Could be Achieved Based on the Operational Architectonics EEG Analysis: A Pilot Feasibility Study. Open Neuroimag J, 10, 69–79. https://doi.org/10.2174/1874440001610010069

47. Fingelkurts, A. A., Fingelkurts, A. A., Bagnato, S., Boccagni, C., & Galardi, G. (2013). Prognostic value of resting-state electroencephalography structure in disentangling vegetative and minimally conscious states: A preliminary study. Neurorehabilitation and Neural Repair, 27(4), 345–354. https://doi.org/10.1177/1545968312469836

48. Formisano, R., Bivona, U., Penta, F., Giustini, M., Buzzi, M. G., Ciurli, P., Matteis, M., Barba, C., Della Vedova, C., Vinicola, V., & Taggi, F. (2005). Early clinical predictive factors during coma recovery. Acta Neurochir Suppl, 93, 201–205. https://doi.org/10.1007/3-211-27577-0_35

49. Formisano, Rita, Jlenia Toppi, Monica Risetti, Marta Aloisi, Marianna Contrada, Paola M. Ciurli, Chiara Falletta Caravasso, u. a. „Language-Related Brain Potentials in Patients With Disorders of Consciousness: A Follow-up Study to Detect "Covert" Language Disorders". Neurorehabilitation and Neural Repair, 23. Mai 2019, 1545968319846123. https://doi.org/10/gf27t5.

50. Gao, Y., Zhang, Y., Li, Z., Ma, L. L., & Yang, J. (2020). Persistent vegetative state after severe cerebral hemorrhage treated with amantadine A retrospective controlled study. Medicine, 99(33). https://doi.org/10.1097/md.0000000000021821

51. Glass, I., Sazbon, L., & Groswasser, Z. (1998). Mapping „cognitive" event-related potentials in prolonged postcoma unawareness state. Clin Electroencephalogr, 29(1), 19–30. https://doi.org/10.1177/155005949802900108

52. Golkowski, D., Merz, K., Mlynarcik, C., Kiel, T., Schorr, B., Lopez-Rolon, A., Lukas, M., Jordan, D., Bender, A., & Ilg, R. (2017). Simultaneous EEG-PET-fMRI measurements in disorders of consciousness: An exploratory study on diagnosis and prognosis. Journal of Neurology, 264(9), 1986–1995. https://doi.org/10.1007/s00415-017-8591-z

53. Gosseries, O., Schnakers, C., Ledoux, D., Vanhaudenhuyse, A., Bruno, M. A., Demertzi, A., Noirhomme, Q., Lehembre, R., Damas, P., Goldman, S., Peeters, E., Moonen, G., & Laureys, S. (2011). Automated EEG entropy measurements in coma, vegetative state/unresponsive wakefulness syndrome and minimally conscious state. Functional Neurology, 26(1), 25–30.

54. Harrison, N., & Wilson, F. C. (2007). Independent living following a „Do Not Resuscitate" order after subarachnoid haemorrhage. Disability and Rehabilitation, 29(4), 347–352. https://doi.org/10.1080/09638280600758956

55. Hauger, S. L., Olafsen, K., Schnakers, C., Andelic, N., Nilsen, K. B., Helseth, E., Funderud, I., Andersson, S., Schanke, A. K., & Lovstad, M. (2017). Cognitive Event-Related Potentials during the Sub-Acute Phase of Severe Traumatic Brain Injury and Their Relationship to Outcome. Journal of Neurotrauma, 34(22), 3124–3133. https://doi.org/10.1089/neu.2017.5062

56. Hermann, B., Brisson, H., Langeron, O., Pyatigorskaya, N., Paquereau, J., Robert, H., Stender, J., Habert, M. O., Naccache, L., & Monsel, A. (2018). Unexpected good

outcome in severe cerebral fat embolism syndrome. Annals of Clinical and Translational Neurology, 5(8), 988–995. https://doi.org/10.1002/acn3.596

57. Hermann, B., Stender, J., Habert, M. O., Kas, A., Denis-Valente, M., Raimondo, F., Pérez, P., Rohaut, B., Sitt, J. D., & Naccache, L. (2021). Multimodal FDG-PET and EEG assessment improves diagnosis and prognostication of disorders of consciousness. Neuroimage Clin, 30, 102601. https://doi.org/10.1016/j.nicl.2021.102600

58. Hu, Y., Yu, F., Wang, C., Yan, X., & Wang, K. (2021). Can Music Influence Patients With Disorders of Consciousness? An Event-Related Potential Study. Front Neurosci, 15, 596636. https://doi.org/10.3389/fnins.2021.596635

59. Illman, N. A., & Crawford, S. (2018). Late-recovery from „permanent“ vegetative state in the context of severe traumatic brain injury: A case report exploring objective and subjective aspects of recovery and rehabilitation. Neuropsychological Rehabilitation, 28(8), 1360–1374. https://doi.org/10.1080/09602011.2017.1313166

60. Jang, S. H., & Lee, H. D. (2020). Recovery of an injured ascending reticular activating system with recovery from a minimally conscious state to normal consciousness in a stroke patient: A diffusion tensor tractography study. Neural Regeneration Research, 15(9), 1767–1768. https://doi.org/10.4103/1673-5374.276362

61. Jang, S. H., Hyun, Y. J., & Lee, H. D. (2016). Recovery of consciousness and an injured ascending reticular activating system in a patient who survived cardiac arrest: A case report. Medicine, 95(26). https://doi.org/10.1097/md.0000000000004041

62. Jang, S. H., Kim, S. H., & Seo, J. P. (2021). Long-term recovery from a minimally responsive state with recovery of an injured ascending reticular activating system: A case report. Medicine (Baltimore), 100(9), e23933. https://doi.org/10.1097/md.0000000000023932

63. Jöhr, J., Halimi, F., Pasquier, J., Pincherle, A., Schiff, N., & Diserens, K. (2020). Recovery in cognitive motor dissociation after severe brain injury: A cohort study. PLoS One, 15(2), e0228474. https://doi.org/10.1371/journal.pone.0228474

64. Keren, O., Sazbon, L., Groswasser, Z., & Shmuel, M. (1994). Follow-up studies of somatosensory evoked potentials and auditory brainstem evoked potentials in patients with post-coma unawareness (PCU) of traumatic brain injury. Brain Injury, 8(3), 239–247. https://doi.org/10.3109/02699059409150975

65. Kim, E. J., Park, J. M., Kim, W. H., Lee, K. L., Kim, H. N., Lee, K. E., Park, J. J., & Ahn, K. O. (2012). A Learning Set Up for Detecting Minimally Conscious State (MCS). Ann Rehabil Med, 36(3), 428–431. https://doi.org/10.5535/arm.2012.36.3.428

66. Kondziella, D., Fisher, P. M., Larsen, V. A., Hauerberg, J., Fabricius, M., Møller, K., & Knudsen, G. M. (2017). Functional MRI for Assessment of the Default Mode Network in Acute Brain Injury. Neurocritical Care, 27(3), 401–406. https://doi.org/10.1007/s12028-017-0407-6

67. Kotchoubey, B., Lang, S., Mezger, G., Schmalohr, D., Schneck, M., Semmler, A., Bostanov, V., & Birbaumer, N. (2005). Information processing in severe disorders of consciousness: Vegetative state and minimally conscious state. Clin

Neurophysiol, 116(10), 2441–2453. https://doi.org/10.1016/j.clinph.2005.03.027

68. Lange-Cosack, H., Riebel, U., Grumme, T., & Schlesener, H. J. (1981). Possibilities and limitations of rehabilitation after traumatic apallic syndrome in children and adolescents. Neuropediatrics, 12(4), 337–365. https://doi.org/10.1055/s-2008-1059665

69. Lee, H. Y., Park, J. H., Kim, A. R., Park, M., & Kim, T. W. (2020). Neurobehavioral recovery in patients who emerged from prolonged disorder of consciousness: A retrospective study. BMC Neurology, 20(1), 198. https://doi.org/10.1186/s12883-020-01758-5

70. Lee, T. M., Savage, J., McKee, H., Flament, M. P., D'Onofrio, S., & Eckert, S. (2013). How do you know when your patient is „waking up": Coma recovery assessment in a complex continuing care setting. Can J Neurosci Nurs, 35(2), 27–33.

71. Lei, L., Liu, K., Yang, Y., Doubliez, A., Hu, X., Xu, Y., & Zhou, Y. (2022). Spatio-temporal analysis of EEG features during consciousness recovery in patients with disorders of consciousness. Clin Neurophysiol, 133, 135–144. https://doi.org/10.1016/j.clinph.2021.08.026

72. Li, L., Kang, X. G., Qi, S., Xu, X. X., Xiong, L. Z., Zhao, G., Yin, H., & Jiang, W. (2015). Brain response to thermal stimulation predicts outcome of patients with chronic disorders of consciousness. Clin Neurophysiol, 126(8), 1539–1547. https://doi.org/10.1016/j.clinph.2014.10.148

73. Li, R., Song, W. Q., Du, J. B., Huo, S., & Shan, G. X. (2015). Connecting the P300 to the diagnosis and prognosis of unconscious patients. Neural Regeneration Research, 10(3), 473–480. https://doi.org/10.4103/1673-5374.153699

74. Li, X., Tan, X., Wang, P., Hu, X., Dong, Y., Zhang, X., & Luo, B. (2020). Chronic disorders of consciousness: A case report with longitudinal evaluation of disease progression using 7 T magnetic resonance imaging. BMC Neurology, 20(1), 396. https://doi.org/10.1186/s12883-020-01973-0

75. Liu, B., Zhang, X., Wang, L., Li, Y., Hou, J., Duan, G., Guo, T., & Wu, D. (2021). Outcome Prediction in Unresponsive Wakefulness Syndrome and Minimally Conscious State by Non-linear Dynamic Analysis of the EEG. Front Neurol, 12, 510424. https://doi.org/10.3389/fneur.2021.510423

76. Logi, F., Pasqualetti, P., & Tomaiuolo, F. (2011). Predict recovery of consciousness in post-acute severe brain injury: The role of EEG reactivity. Brain Injury, 25(10), 972–979. https://doi.org/10.3109/02699052.2011.589794

77. Løvstad, M., Andelic, N., Knoph, R., Jerstad, T., Anke, A., Skandsen, T., Hauger, S. L., Giacino, J. T., Røe, C., & Schanke, A. K. (2014). Rate of disorders of consciousness in a prospective population-based study of adults with traumatic brain injury. J Head Trauma Rehabil, 29(5), E31-43. https://doi.org/10.1097/htr.0000000000000017

78. Meiron, O., Barron, J., David, J., & Jaul, E. (2021). Neural reactivity parameters of awareness predetermine one-year survival in patients with disorders of consciousness. Brain Injury, 35(4), 453–459. https://doi.org/10.1080/02699052.2021.1879397

79. Nekrasova, J., Kanarskii, M., Borisov, I., Pradhan, P., Shunenkov, D., Vorobiev, A., Smirnova, M., Pasko, V., Petrova, M. V., Luginina, E., & Pryanikov, I. (2021). One-

Year Demographical and Clinical Indices of Patients with Chronic Disorders of Consciousness. Brain Sci, 11(5). https://doi.org/10.3390/brainsci11050650

80. Oknina, L, O Zaitsev, E Masherow, M Kopachka, und E Sharova. „The Use of Event-Related Potentials for Predicting the Degree of Mental Recovery in Patients with Severe Brain Injury – A Prospective Study“. Journal of Advances in Medicine and Medical Research 27, Nr. 4 (6. August 2018): 1–13. https://doi.org/10/ghdmdv.

81. Pan, J., Xie, Q., Lin, Q., Huang, H., Fei, W., Yu, R., & Li, Y. (2017). Prediction of consciousness recovery in patients with disorder of consciousness using Brain-computer Interface. International IEEE/EMBS Conference on Neural Engineering, NER, 419–423. https://doi.org/10.1109/NER.2017.8008379

82. Pan, J., Xie, Q., Qin, P., Chen, Y., He, Y., Huang, H., Wang, F., Ni, X., Cichocki, A., Yu, R., & Li, Y. (2020). Prognosis for patients with cognitive motor dissociation identified by brain-computer interface. Brain, 143(4), 1177–1189. https://doi.org/10.1093/brain/awaa026

83. Pascarella, A., Trojano, L., Loreto, V., Bilo, L., Moretta, P., & Estraneo, A. (2016). Long-term outcome of patients with disorders of consciousness with and without epileptiform activity and seizures: A prospective single centre cohort study. Journal of Neurology, 263(10), 2048–2056. https://doi.org/10.1007/s00415-016-8232-y

84. Perez, P., Valente, M., Hermann, B., Sitt, J., Faugeras, F., Demeret, S., Rohaut, B., & Naccache, L. (2021). Auditory Event-Related “Global Effect” Predicts Recovery of Overt Consciousness. Frontiers in Neurology, 11. https://doi.org/10.3389/fneur.2020.588232

85. Pham, K., Kramer, M. E., Slomine, B. S., & Suskauer, S. J. (2014). Emergence to the conscious state during inpatient rehabilitation after traumatic brain injury in children and young adults: A case series. J Head Trauma Rehabil, 29(5), E44-8. https://doi.org/10.1097/htr.0000000000000021

86. Pignat, J. M., Mauron, E., Jöhr, J., Gilart de Keranflec’h, C., Van De Ville, D., Preti, M. G., Meskaldji, D. E., Hömberg, V., Laureys, S., Draganski, B., Frackowiak, R., & Diserens, K. (2016). Outcome Prediction of Consciousness Disorders in the Acute Stage Based on a Complementary Motor Behavioural Tool. PLoS One, 11(6), e0156882. https://doi.org/10.1371/journal.pone.0156881

87. Pistoia, F., Sacco, S., Palmirotta, R., Onorati, P., Carolei, A., & Sarà, M. (2008). Mismatch of neurophysiological findings in partial recovery of consciousness: A case report. Brain Injury, 22(7–8), 633–637. https://doi.org/10.1080/02699050802189692

88. Qin, P., Di, H., Yan, X., Yu, S., Yu, D., Laureys, S., & Weng, X. (2008). Mismatch negativity to the patient’s own name in chronic disorders of consciousness. Neuroscience Letters, 448(1), 24–28. https://doi.org/10.1016/j.neulet.2008.10.028

89. Qin, P., Wu, X., Duncan, N. W., Bao, W., Tang, W., Zhang, Z., Hu, J., Jin, Y., Wu, X., Gao, L., Lu, L., Guan, Y., Lane, T., Huang, Z., Bodien, Y. G., Giacino, J. T., Mao, Y., & Northoff, G. (2015). GABAA receptor deficits predict recovery in patients with disorders of consciousness: A preliminary multimodal [(11) C]Flumazenil PET

and fMRI study. Human Brain Mapping, 36(10), 3867–3877. https://doi.org/10.1002/hbm.22883

90. Qin, P., Wu, X., Huang, Z., Duncan, N. W., Tang, W., Wolff, A., Hu, J., Gao, L., Jin, Y., Wu, X., Zhang, J., Lu, L., Wu, C., Qu, X., Mao, Y., Weng, X., Zhang, J., & Northoff, G. (2015). How are different neural networks related to consciousness? Annals of Neurology, 78(4), 594–605. https://doi.org/10.1002/ana.24479

91. Risetti, M., Formisano, R., Toppi, J., Quitadamo, L. R., Bianchi, L., Astolfi, L., Cincotti, F., & Mattia, D. (2013). On ERPs detection in disorders of consciousness rehabilitation. Frontiers in Human Neuroscience, 7, 775. https://doi.org/10.3389/fnhum.2013.00775

92. Rohaut, B., Faugeras, F., Chausson, N., King, J. R., Karoui, I. E., Cohen, L., & Naccache, L. (2015). Probing ERP correlates of verbal semantic processing in patients with impaired consciousness. Neuropsychologia, 66, 279–292. https://doi.org/10.1016/j.neuropsychologia.2014.10.014

93. Rousseau, M. C., Confort-Gouny, S., Catala, A., Graperon, J., Blaya, J., Soulier, E., Viout, P., Galanaud, D., Le Fur, Y., Cozzone, P. J., & Ranjeva, J. P. (2008). A MRS-MRI-fMRI exploration of the brain. Impact of long-lasting persistent vegetative state. Brain Injury, 22(2), 123–134. https://doi.org/10.1080/02699050801895414

94. Sancisi, E., Battistini, A., Di Stefano, C., Simoncini, L., Simoncini, L., Montagna, P., & Piperno, R. (2009). Late recovery from post-traumatic vegetative state. Brain Injury, 23(2), 163–166. https://doi.org/10.1080/02699050802660445

95. Schorl, M., Valerius-Kukula, S. J., & Kemmer, T. P. (2014). Median-evoked somatosensory potentials in severe brain injury: Does initial loss of cortical potentials exclude recovery? Clinical Neurology and Neurosurgery, 123, 25–33. https://doi.org/10.1016/j.clineuro.2014.05.004

96. Schorr, B., Schlee, W., Arndt, M., & Bender, A. (2016). Coherence in resting-state EEG as a predictor for the recovery from unresponsive wakefulness syndrome. Journal of Neurology, 263(5), 937–953. https://doi.org/10.1007/s00415-016-8084-5

97. Song, Ming, Yi Yang, Jianghong He, Zhengyi Yang, Shan Yu, Qiuyou Xie, Xiaoyu Xia, u. a. „Prognostication of Chronic Disorders of Consciousness Using Brain Functional Networks and Clinical Characteristics“. ELife 7 (14. August 2018). https://doi.org/10/gd28cb.

98. Spataro, R., Heilinger, A., Allison, B. Z., De Cicco, D., Marchese, S., Gregoretti, C., La Bella, V., & Guger, C. (2018). Preserved somatosensory discrimination predicts consciousness recovery in unresponsive wakefulness syndrome. 129(6), 1130–1136. https://doi.org/10.1016/j.clinph.2018.02.131

99. Steinbock, B. (1989). Recovery from persistent vegetative state? The case of Carrie Coons. Hastings Cent Rep, 19(4), 14–15. https://doi.org/10.2307/3562293

100. Stender, J., Gosseries, O., Bruno, M. A., Charland-Verville, V., Vanhaudenhuyse, A., Demertzi, A., Chatelle, C., Thonnard, M., Thibaut, A., Heine, L., Soddu, A., Boly, M., Schnakers, C., Gjedde, A., & Laureys, S. (2014). Diagnostic precision of PET imaging and functional MRI in disorders of consciousness: A clinical validation

study. Lancet, 384(9942), 514–522. https://doi.org/10.1016/s0140-6736(14)60042-7

101. Steppacher, I., Kaps, M., & Kissler, J. (2014). Will time heal? A long-term follow-up of severe disorders of consciousness. Annals of Clinical and Translational Neurology, 1(6), 401–408. https://doi.org/10.1002/acn3.63

102. Steppacher, I., Kaps, M., & Kissler, J. (2016). Against the odds: A case study of recovery from coma after devastating prognosis. Annals of Clinical and Translational Neurology, 3(1), 61–65. https://doi.org/10.1002/acn3.269

103. Stoica, S. I., Chiparus, C. E., Lapadat, M. V., Nohai, I. M., Dumitrascu, A., Andone, I., Popescu, C., & Onose, G. (2020). Clinical-imagistic and rehabilitation features in a young patient traumatized by road accident- with vegetative state and evolution towards a state of minimum consciousness at 6 months after the accident. Balneo Research Journal, 11(4), 551–553. https://doi.org/10.12680/balneo.2020.402

104. Talar, J. (2002). Rehabilitation outcome in a patient awakened from prolonged coma. Med Sci Monit, 8(4), Cs31-8.

105. Tamashiro, M., Cozzo, D., Mattei, M., Salierno, F., Rivas, M. E., Alzúa, O., Olmos, L., Bonamico, L., & Leiguarda, R. (2012). Early motor predictors of recovery in patients with severe traumatic brain injury. Brain Injury, 26(7–8), 921–926. https://doi.org/10.3109/02699052.2012.661910

106. Tan, X., Gao, J., Zhou, Z., Wei, R., Gong, T., Wu, Y., Liu, K., He, F., Wang, J., Li, J., Zhang, X., Pan, G., & Luo, B. (2017). Spontaneous Recovery from Unresponsive Wakefulness Syndrome to a Minimally Conscious State: Early Structural Changes Revealed by 7-T Magnetic Resonance Imaging. Frontiers in Neurology, 8, 741. https://doi.org/10.3389/fneur.2017.00741

107. Thibaut, A., Panda, R., Annen, J., Sanz, L. R. D., Naccache, L., Martial, C., Chatelle, C., Aubinet, C., Bonin, E. A. C., Barra, A., Briand, M. M., Cecconi, B., Wannez, S., Stender, J., Laureys, S., & Gosseries, O. (2021). Preservation of Brain Activity in Unresponsive Patients Identifies MCS Star. Ann Neurol, 90(1), 89–100. https://doi.org/10.1002/ana.26095

108. van Erp, W. S., Aben, A. M. L., Lavrijsen, J. C. M., Vos, P. E., Laureys, S., & Koopmans, R. (2019). Unexpected emergence from the vegetative state: Delayed discovery rather than late recovery of consciousness. Journal of Neurology, 266(12), 3144–3149. https://doi.org/10.1007/s00415-019-09542-2

109. Vanhaudenhuyse, A., Giacino, J., Schnakers, C., Kalmar, K., Smart, C., Bruno, M. A., Gosseries, O., Moonen, G., & Laureys, S. (2008). BLink to visual threat does not herald consciousness in the vegetative state. Neurology, 71(17), 1374–1375. https://doi.org/10.1212/01.wnl.0000320110.70134.59

110. Vogel, D., Markl, A., Yu, T., Kotchoubey, B., Lang, S., & Müller, F. (2013). Can mental imagery functional magnetic resonance imaging predict recovery in patients with disorders of consciousness? Archives of Physical Medicine and Rehabilitation, 94(10), 1891–1898. https://doi.org/10.1016/j.apmr.2012.11.053

111. Wang, F., Di, H., Hu, X., Jing, S., Thibaut, A., Di Perri, C., Huang, W., Nie, Y., Schnakers, C., & Laureys, S. (2015). Cerebral response to subject's own name

showed high prognostic value in traumatic vegetative state. BMC Med, 13, 83. https://doi.org/10.1186/s12916-015-0330-7

112. Wang, F., Hu, N., Hu, X., Jing, S., Heine, L., Thibaut, A., Huang, W., Yan, Y., Wang, J., Schnakers, C., Laureys, S., & Di, H. (2019). Detecting Brain Activity Following a Verbal Command in Patients With Disorders of Consciousness. Frontiers in Neuroscience, 13, 976. https://doi.org/10.3389/fnins.2019.00976

113. Wang, X. Y., Wu, H. Y., Lu, H. T., Huang, T. T., Zhang, H., & Zhang, T. (2017). Assessment of mismatch negativity and P300 response in patients with disorders of consciousness. European Review for Medical and Pharmacological Sciences, 21(21), 4896–4906.

114. Wang, X., Zhang, H., Zhang, X., Sun, X., & Zhang, T. (2015). Assessing consciousness with auditory event-related potential during coma recovery: A case study. Neuroreport, 26(2), 50–56. https://doi.org/10.1097/wnr.0000000000000288

115. Weiss, N., Tadie, J. M., Faugeras, F., Diehl, J. L., Fagon, J. Y., & Guerot, E. (2012). Can fast-component of nystagmus on caloric vestibulo-ocular responses predict emergence from vegetative state in ICU? Journal of Neurology, 259(1), 70–76. https://doi.org/10.1007/s00415-011-6120-z

116. Wijnen, V. J. M., Heutink, M., Boxtel, G. J. M. v, Eilander, H. J., & Gelder, B. d. (2006). Autonomic reactivity to sensory stimulation is related to consciousness level after severe traumatic brain injury. Clinical Neurophysiology, 117(8), 1794–1807. https://doi.org/10.1016/j.clinph.2006.03.005

117. Wijnen, V. J., Eilander, H. J., de Gelder, B., & van Boxtel, G. J. (2014). Repeated measurements of the auditory oddball paradigm is related to recovery from the vegetative state. J Clin Neurophysiol, 31(1), 65–80. https://doi.org/10.1097/01.wnp.0000436894.17749.0c

118. Wu, Min, Wang-Xiao Bao, Jie Zhang, Yang-Fan Hu, Jian Gao, und Ben-Yan Luo. „Effect of Acoustic Stimuli in Patients with Disorders of Consciousness: A Quantitative Electroencephalography Study“. Neural Regeneration Research 13, Nr. 11 (2018): 1900. https://doi.org/10/ghdmds.

119. Wu, X., Zou, Q., Hu, J., Tang, W., Mao, Y., Gao, L., Zhu, J., Jin, Y., Wu, X., Lu, L., Zhang, Y., Zhang, Y., Dai, Z., Gao, J. H., Weng, X., Zhou, L., Northoff, G., Giacino, J. T., He, Y., & Yang, Y. (2015). Intrinsic Functional Connectivity Patterns Predict Consciousness Level and Recovery Outcome in Acquired Brain Injury. J Neurosci, 35(37), 12932–12946. https://doi.org/10.1523/jneurosci.0415-15.2015

120. Xiao, J., Xie, Q., Lin, Q., Yu, T., Yu, R., & Li, Y. (2018). Assessment of Visual Pursuit in Patients With Disorders of Consciousness Based on a Brain-Computer Interface. IEEE Trans Neural Syst Rehabil Eng, 26(6), 1141–1151. https://doi.org/10.1109/tnsre.2018.2835813

121. Xie, Q., Pan, J., Chen, Y., He, Y., Ni, X., Zhang, J., … Yu, R. (2018). A gaze-independent audiovisual brain-computer Interface for detecting awareness of patients with disorders of consciousness. BMC Neurology, 18(1), 144. https://doi.org/10/ggn4ck

122. Xu, C., Zou, J., He, F., Wen, X., Li, J., Gao, J., Ding, N., & Luo, B. (2021). Neural Tracking of Sound Rhythms Correlates With Diagnosis, Severity, and Prognosis

of Disorders of Consciousness. Frontiers in Neuroscience, 15. https://doi.org/10.3389/fnins.2021.646542

123. Yelden, K., Duport, S., James, L. M., Kempny, A., Farmer, S. F., Leff, A. P., & Playford, E. D. (2018). Late recovery of awareness in prolonged disorders of consciousness -a cross-sectional cohort study. Disability and Rehabilitation, 40(20), 2433–2438. https://doi.org/10.1080/09638288.2017.1339209

124. Yu, Y., Meng, F., Zhang, L., Liu, X., Wu, Y., Chen, S., Tan, X., Li, X., Kuang, S., Sun, Y., & Luo, B. (2021). A multi-domain prognostic model of disorder of consciousness using resting-state fMRI and laboratory parameters. Brain Imaging Behav, 15(4), 1966–1976. https://doi.org/10.1007/s11682-020-00390-7

125. Zhang, L., Luo, L., Zhou, Z., Xu, K., Zhang, L., Liu, X., Tan, X., Zhang, J., Ye, X., Gao, J., & Luo, B. (2018). Functional Connectivity of Anterior Insula Predicts Recovery of Patients With Disorders of Consciousness. Frontiers in Neurology, 9, 1024. https://doi.org/10.3389/fneur.2018.01024

126. Zhang, Y., Li, R., Du, J., Huo, S., Hao, J., & Song, W. (2017). Coherence in P300 as a predictor for the recovery from disorders of consciousness. Neuroscience Letters, 653, 332–336. https://doi.org/10.1016/j.neulet.2017.06.013

127. Zou, Q., Wu, X., Hu, J., Tang, W., Mao, Y., Zhu, J., Lu, L., Zhang, Y., & Gao, J. H. (2017). Longitudinal recovery of local neuronal activity and consciousness level in acquired brain injury. Human Brain Mapping, 38(7), 3579–3591. https://doi.org/10.1002/hbm.23611

Table S1 - Number of patients by observation time bins

| | Time since onset at follow-up examination, years | n | % regained consciousness | Median age, years |
|---|---|---|---|---|
| **Sex** | | | | |
| Male | <1 | 1,165 | 39.06 | 44.00 |
| | 1-5 | 763 | 34.08 | 44.00 |
| | >5 | 101 | 34.65 | 35.00 |
| Female | <1 | 532 | 31.58 | 50.00 |
| | 1-5 | 476 | 31.72 | 48.00 |
| | >5 | 48 | 41.67 | 45.00 |
| **Diagnosis** | | | | |
| UWS | <1 | 1,149 | 23.06 | 46.00 |
| | 1-5 | 749 | 18.02 | 46.00 |
| | >5 | 80 | 18.75 | 37.50 |
| MCS | <1 | 678 | 60.62 | 46.00 |
| | 1-5 | 555 | 55.50 | 44.00 |
| | >5 | 79 | 59.49 | 35.50 |
| MCS- | <1 | 315 | 51.43 | 48.00 |
| | 1-5 | 323 | 57.28 | 47.00 |
| | >5 | 34 | 44.12 | 43.00 |
| MCS+ | <1 | 84 | 64.29 | 43.72 |
| | 1-5 | 94 | 69.15 | 44.75 |
| | >5 | 27 | 88.89 | 31.00 |
| **Etiology** | | | | |
| TBI | <1 | 866 | 45.84 | 37.00 |
| | 1-5 | 574 | 43.38 | 35.50 |
| | >5 | 83 | 44.58 | 30.00 |
| non-TBI | <1 | 960 | 28.96 | 52.00 |
| | 1-5 | 730 | 26.58 | 50.00 |
| | >5 | 76 | 32.89 | 43.00 |
| anoxic | <1 | 283 | 18.02 | 50.00 |
| | 1-5 | 308 | 15.26 | 45.00 |
| | >5 | 44 | 18.18 | 43.50 |
| vascular | <1 | 447 | 32.66 | 54.00 |
| | 1-5 | 262 | 35.11 | 55.00 |
| | >5 | 9 | 33.33 | 45.00 |

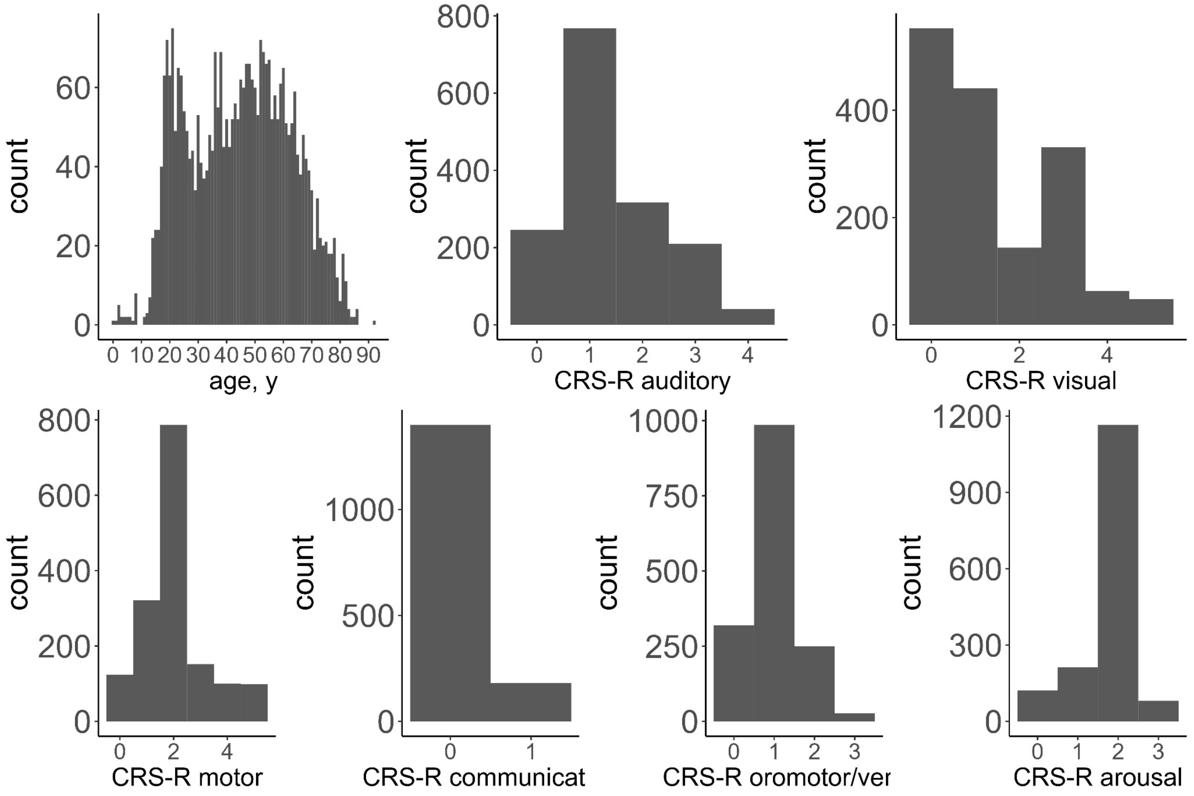


Figure S1 – Distribution of the patients' age and scores on the CSR-R scales.

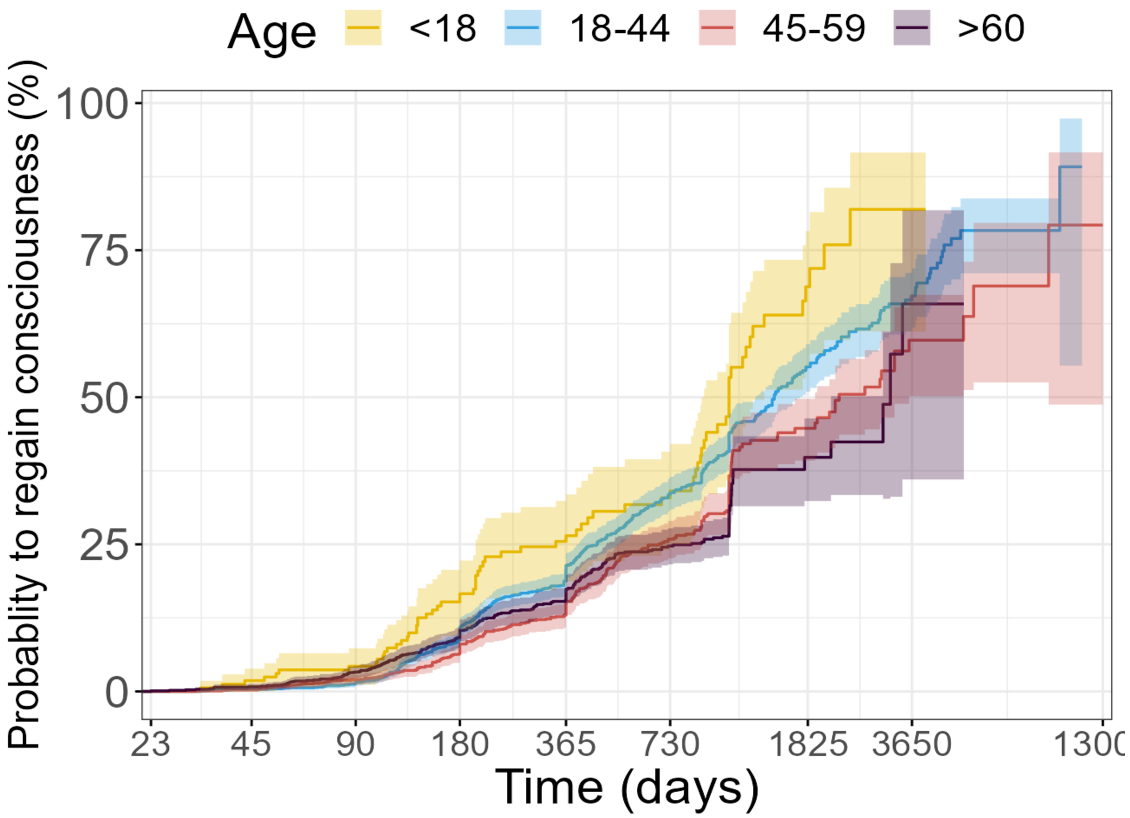


Figure S2 – Kaplan-Meier curves stratified by age groups. In contrast to the main analyses, patients who died at the time of follow-up examination are not excluded.

# Multivariable analyses

## Original without cluster correction

Table S2 - Diagnosis, 2 etiologies

| Effect | n | b | SE | z | p | HR | CI lower | CI upper |
|---|---|---|---|---|---|---|---|---|
| | | | | **90 days** | | | | |
| **Age** | 3082 | 0.02 | 0.007 | 2.981 | 0.003 | 1.02 | 1.01 | 1.03 |
| **Sex** | | | | | | | | |
| Male | 2028 | | | | | | | |
| Female | 1054 | -0.248 | 0.272 | -0.911 | 0.362 | 0.78 | 0.46 | 1.33 |
| **Diagnosis** | | | | | | | | |
| MCS | 1260 | | | | | | | |
| UWS | 1822 | -0.8 | 0.253 | -3.158 | 0.002 | 0.45 | 0.27 | 0.74 |
| **Etiology** | | | | | | | | |
| non-TBI | 1667 | | | | | | | |
| TBI | 1415 | 0.182 | 0.211 | 0.86 | 0.39 | 1.2 | 0.79 | 1.81 |
| | | | | **180 days** | | | | |
| **Age** | 3082 | 0.007 | 0.003 | 2.935 | 0.003 | 1.01 | 1 | 1.01 |
| **Sex** | | | | | | | | |
| Male | 2028 | | | | | | | |
| Female | 1054 | -0.116 | 0.119 | -0.972 | 0.331 | 0.89 | 0.71 | 1.12 |
| **Diagnosis** | | | | | | | | |
| MCS | 1260 | | | | | | | |
| UWS | 1822 | -0.938 | 0.1 | -9.375 | <.001 | 0.39 | 0.32 | 0.48 |
| **Etiology** | | | | | | | | |
| non-TBI | 1667 | | | | | | | |
| TBI | 1415 | 0.603 | 0.105 | 5.767 | <.001 | 1.83 | 1.49 | 2.24 |
| | | | | **365 days** | | | | |
| **Age** | 3082 | 0.005 | 0.002 | 2.435 | 0.015 | 1 | 1 | 1.01 |
| **Sex** | | | | | | | | |
| Male | 2028 | | | | | | | |
| Female | 1054 | -0.279 | 0.102 | -2.735 | 0.006 | 0.76 | 0.62 | 0.92 |
| **Diagnosis** | | | | | | | | |
| MCS | 1260 | | | | | | | |
| UWS | 1822 | -1.032 | 0.088 | -11.73 | <.001 | 0.36 | 0.3 | 0.42 |

| | | | | | | | | |
|---|---|---|---|---|---|---|---|---|
| **Etiology** | | | | | | | | |
| non-TBI | 1667 | | | | | | | |
| TBI | 1415 | 0.451 | 0.084 | 5.343 | <.001 | 1.57 | 1.33 | 1.85 |
| | | | | **1825 days** | | | | |
| **Age** | 3082 | 0.003 | 0.002 | 1.464 | 0.143 | 1 | 1 | 1.01 |
| **Sex** | | | | | | | | |
| Male | 2028 | | | | | | | |
| Female | 1054 | -0.134 | 0.074 | -1.812 | 0.07 | 0.87 | 0.76 | 1.01 |
| **Diagnosis** | | | | | | | | |
| MCS | 1260 | | | | | | | |
| UWS | 1822 | -1.1 | 0.074 | -14.85 | <.001 | 0.33 | 0.29 | 0.38 |
| **Etiology** | | | | | | | | |
| non-TBI | 1667 | | | | | | | |
| TBI | 1415 | 0.342 | 0.063 | 5.437 | <.001 | 1.41 | 1.24 | 1.59 |
| | | | | **3650 days** | | | | |
| **Age** | 3082 | 0.002 | 0.002 | 1.311 | 0.19 | 1 | 1 | 1.01 |
| **Sex** | | | | | | | | |
| Male | 2028 | | | | | | | |
| Female | 1054 | -0.119 | 0.071 | -1.671 | 0.095 | 0.89 | 0.77 | 1.02 |
| **Diagnosis** | | | | | | | | |
| MCS | 1260 | | | | | | | |
| UWS | 1822 | -1.099 | 0.069 | -15.97 | <.001 | 0.33 | 0.29 | 0.38 |
| **Etiology** | | | | | | | | |
| non-TBI | 1667 | | | | | | | |
| TBI | 1415 | 0.316 | 0.063 | 4.986 | <.001 | 1.37 | 1.21 | 1.55 |

Table S3 - Diagnosis, 3 etiologies

| Effect | n | b | SE | z | p | HR | CI lower | CI upper |
|---|---|---|---|---|---|---|---|---|
| | | | | **90 days** | | | | |
| **Age** | 2693 | 0.015 | 0.008 | 1.881 | 0.06 | 1.01 | 1 | 1.03 |
| **Sex** | | | | | | | | |
| Male | 1808 | | Female | 885 | | | | |
| Female | 885 | -0.22 | 0.258 | -0.853 | 0.394 | 0.8 | 0.48 | 1.33 |
| **Diagnosis** | | | | | | | | |
| MCS | 1084 | | | | | | | |
| UWS | 1609 | -0.389 | 0.254 | -1.535 | 0.125 | 0.68 | 0.41 | 1.11 |

| | | | | | | | | |
|---|---|---|---|---|---|---|---|---|
| **Etiology** | | | | | | | | |
| Anoxic | 596 | | | | | | | |
| TBI | 1415 | 1.082 | 1.121 | 0.965 | 0.335 | 2.95 | 0.33 | 26.58 |
| Vascular | 682 | 1.091 | 1.144 | 0.954 | 0.34 | 2.98 | 0.32 | 28.02 |
| | | | **180 days** | | | | | |
| **Age** | 2693 | 0.002 | 0.004 | 0.597 | 0.55 | 1 | 0.99 | 1.01 |
| **Sex** | | | | | | | | |
| Male | 1808 | | | | | | | |
| Female | 885 | -0.077 | 0.132 | -0.584 | 0.559 | 0.93 | 0.71 | 1.2 |
| **Diagnosis** | | | | | | | | |
| MCS | 1084 | | | | | | | |
| UWS | 1609 | -0.867 | 0.122 | -7.126 | <.001 | 0.42 | 0.33 | 0.53 |
| **Etiology** | | | | | | | | |
| Anoxic | 596 | | | | | | | |
| TBI | 1415 | 1.215 | 0.228 | 5.32 | <.001 | 3.37 | 2.15 | 5.27 |
| Vascular | 682 | 0.817 | 0.233 | 3.509 | <.001 | 2.26 | 1.43 | 3.57 |
| | | | **365 days** | | | | | |
| **Age** | 2693 | 0.001 | 0.003 | 0.279 | 0.78 | 1 | 1 | 1.01 |
| **Sex** | | | | | | | | |
| Male | 1808 | | | | | | | |
| Female | 885 | -0.273 | 0.083 | -3.308 | 0.001 | 0.76 | 0.65 | 0.89 |
| **Diagnosis** | | | | | | | | |
| MCS | 1084 | | | | | | | |
| UWS | 1609 | -0.961 | 0.089 | -10.85 | <.001 | 0.38 | 0.32 | 0.46 |
| **Etiology** | | | | | | | | |
| Anoxic | 596 | | | | | | | |
| TBI | 1415 | 1.078 | 0.151 | 7.138 | <.001 | 2.94 | 2.19 | 3.95 |
| Vascular | 682 | 0.907 | 0.167 | 5.42 | <.001 | 2.48 | 1.78 | 3.44 |
| | | | **1825 days** | | | | | |
| **Age** | 2693 | -0.001 | 0.002 | -0.602 | 0.547 | 1 | 0.99 | 1 |
| **Sex** | | | | | | | | |
| Male | 1808 | | | | | | | |
| Female | 885 | -0.128 | 0.089 | -1.449 | 0.147 | 0.88 | 0.74 | 1.05 |
| **Diagnosis** | | | | | | | | |
| MCS | 1084 | | | | | | | |
| UWS | 1609 | -1.069 | 0.085 | -12.58 | <.001 | 0.34 | 0.29 | 0.41 |

| | | | | | | | | |
|---|---|---|---|---|---|---|---|---|
| **Etiology** | | | | | | | | |
| Anoxic | 596 | | | | | | | |
| TBI | 1415 | 0.859 | 0.123 | 6.992 | <.001 | 2.36 | 1.86 | 3 |
| Vascular | 682 | 0.825 | 0.13 | 6.369 | <.001 | 2.28 | 1.77 | 2.94 |
| | | | | **3650 days** | | | | |
| **Age** | 2693 | -0.002 | 0.002 | -0.756 | 0.45 | 1 | 0.99 | 1 |
| **Sex** | | | | | | | | |
| Male | 1808 | | | | | | | |
| Female | 885 | -0.118 | 0.078 | -1.519 | 0.129 | 0.89 | 0.76 | 1.03 |
| **Diagnosis** | | | | | | | | |
| MCS | 1084 | | | | | | | |
| UWS | 1609 | -1.063 | 0.073 | -14.51 | <.001 | 0.35 | 0.3 | 0.4 |
| **Etiology** | | | | | | | | |
| Anoxic | 596 | | | | | | | |
| TBI | 1415 | 0.819 | 0.114 | 7.162 | <.001 | 2.27 | 1.81 | 2.84 |
| Vascular | 682 | 0.795 | 0.113 | 7.027 | <.001 | 2.21 | 1.77 | 2.76 |

Table S4 - MCS+-, 2 etiologies

| Effect | n | b | SE | z | p | HR | CI lower | CI upper |
|---|---|---|---|---|---|---|---|---|
| | | | | **90 days** | | | | |
| **Age** | 842 | 0.007 | 0.012 | 0.64 | 0.522 | 1.01 | 0.98 | 1.03 |
| **Sex** | | | | | | | | |
| Male | 549 | | | | | | | |
| Female | 293 | -0.024 | 0.443 | -0.053 | 0.958 | 0.98 | 0.41 | 2.33 |
| **Diagnosis** | | | | | | | | |
| MCS- | 657 | | | | | | | |
| MCS+ | 185 | -0.297 | 1.901 | -0.156 | 0.876 | 0.74 | 0.02 | 30.82 |
| **Etiology** | | | | | | | | |
| non-TBI | 419 | | | | | | | |
| TBI | 423 | 0.071 | 0.313 | 0.226 | 0.821 | 1.07 | 0.58 | 1.98 |
| | | | | **180 days** | | | | |
| **Age** | 842 | 0.016 | 0.005 | 3.11 | 0.002 | 1.02 | 1.01 | 1.03 |
| **Sex** | | | | | | | | |
| Male | 549 | | | | | | | |
| Female | 293 | -0.336 | 0.205 | -1.64 | 0.101 | 0.71 | 0.48 | 1.07 |

| | | | | | | | | |
|---|---|---|---|---|---|---|---|---|
| **Diagnosis** | | | | | | | | |
| MCS- | 657 | | | | | | | |
| MCS+ | 185 | -0.464 | 0.242 | -1.919 | 0.055 | 0.63 | 0.39 | 1.01 |
| **Etiology** | | | | | | | | |
| non-TBI | 419 | | | | | | | |
| TBI | 423 | 0.249 | 0.18 | 1.389 | 0.165 | 1.28 | 0.9 | 1.82 |
| | | | **365 days** | | | | | |
| **Age** | 842 | 0.012 | 0.004 | 3.049 | 0.002 | 1.01 | 1 | 1.02 |
| **Sex** | | | | | | | | |
| Male | 549 | | | | | | | |
| Female | 293 | -0.407 | 0.14 | -2.917 | 0.004 | 0.67 | 0.51 | 0.87 |
| **Diagnosis** | | | | | | | | |
| MCS- | 657 | | | | | | | |
| MCS+ | 185 | 0.158 | 0.159 | 0.997 | 0.319 | 1.17 | 0.86 | 1.6 |
| **Etiology** | | | | | | | | |
| non-TBI | 419 | | | | | | | |
| TBI | 423 | -0.045 | 0.134 | -0.339 | 0.735 | 0.96 | 0.74 | 1.24 |
| | | | **1825 days** | | | | | |
| **Age** | 842 | 0.009 | 0.002 | 3.729 | <.001 | 1.01 | 1 | 1.01 |
| **Sex** | | | | | | | | |
| Male | 549 | | | | | | | |
| Female | 293 | -0.268 | 0.104 | -2.586 | 0.01 | 0.76 | 0.62 | 0.94 |
| **Diagnosis** | | | | | | | | |
| MCS- | 657 | | | | | | | |
| MCS+ | 185 | 0.019 | 0.11 | 0.17 | 0.865 | 1.02 | 0.82 | 1.26 |
| **Etiology** | | | | | | | | |
| non-TBI | 419 | | | | | | | |
| TBI | 423 | -0.088 | 0.107 | -0.822 | 0.411 | 0.92 | 0.74 | 1.13 |
| | | | **3650 days** | | | | | |
| **Age** | 842 | 0.009 | 0.003 | 3.285 | 0.001 | 1.01 | 1 | 1.01 |
| **Sex** | | | | | | | | |
| Male | 549 | | | | | | | |
| Female | 293 | -0.259 | 0.107 | -2.424 | 0.015 | 0.77 | 0.63 | 0.95 |
| **Diagnosis** | | | | | | | | |
| MCS- | 657 | | | | | | | |
| MCS+ | 185 | 0.061 | 0.102 | 0.595 | 0.552 | 1.06 | 0.87 | 1.3 |

| | | | | | | | | |
|---|---|---|---|---|---|---|---|---|
| **Etiology** | | | | | | | | |
| non-TBI | 419 | | | | | | | |
| TBI | 423 | -0.116 | 0.104 | -1.117 | 0.264 | 0.89 | 0.73 | 1.09 |

Table S5 - CRS-R scales, 2 etiologies

| Effect | n | b | SE | z | p | HR | CI lower | CI upper |
|---|---|---|---|---|---|---|---|---|
| | | | | **90 days** | | | | |
| **Age** | 1558 | 0.03 | 0.012 | 2.445 | 0.014 | 1.03 | 1.01 | 1.06 |
| **Sex** | | | | | | | | |
| Male | 973 | Female | 585 | | | | | |
| Female | 585 | -0.311 | 0.397 | -0.784 | 0.433 | 0.73 | 0.34 | 1.59 |
| **CRS auditory scale** | 1558 | 0.4 | 0.247 | 1.617 | 0.106 | 1.49 | 0.92 | 2.42 |
| **CRS visual scale** | 1558 | 0.35 | 0.174 | 2.015 | 0.044 | 1.42 | 1.01 | 2 |
| **CRS motor scale** | 1558 | -0.027 | 0.149 | -0.181 | 0.857 | 0.97 | 0.73 | 1.3 |
| **CRS communication scale** | 1558 | -0.991 | 1.771 | -0.559 | 0.576 | 0.37 | 0.01 | 11.94 |
| **CRS oromotor/verbal scale** | 1558 | 0.609 | 0.246 | 2.48 | 0.013 | 1.84 | 1.14 | 2.97 |
| **CRS arousal scale** | 1558 | -0.653 | 0.227 | -2.879 | 0.004 | 0.52 | 0.33 | 0.81 |
| **Etiology** | | | | | | | | |
| non-TBI | 951 | | | | | | | |
| TBI | 607 | -0.153 | 0.455 | -0.336 | 0.737 | 0.86 | 0.35 | 2.09 |
| | | | | **180 days** | | | | |
| **Age** | 1558 | 0.019 | 0.005 | 3.633 | <.001 | 1.02 | 1.01 | 1.03 |
| **Sex** | | | | | | | | |
| Male | 973 | | | | | | | |
| Female | 585 | -0.452 | 0.196 | -2.311 | 0.021 | 0.64 | 0.43 | 0.93 |
| CRS auditory scale | 1558 | 0.298 | 0.115 | 2.585 | 0.01 | 1.35 | 1.07 | 1.69 |
| CRS visual scale | 1558 | 0.162 | 0.09 | 1.805 | 0.071 | 1.18 | 0.99 | 1.4 |
| CRS motor scale | 1558 | 0.394 | 0.079 | 5.006 | <.001 | 1.48 | 1.27 | 1.73 |
| CRS communication scale | 1558 | -0.413 | 0.192 | -2.153 | 0.031 | 0.66 | 0.45 | 0.96 |
| CRS oromotor/verbal scale | 1558 | 0.491 | 0.155 | 3.165 | 0.002 | 1.63 | 1.21 | 2.22 |
| CRS arousal scale | 1558 | -0.566 | 0.134 | -4.21 | <.001 | 0.57 | 0.44 | 0.74 |
| **Etiology** | | | | | | | | |
| non-TBI | 951 | | | | | | | |
| TBI | 607 | 0.352 | 0.192 | 1.831 | 0.067 | 1.42 | 0.98 | 2.07 |
| | | | | **365 days** | | | | |

| | | | | | | | | |
|---|---|---|---|---|---|---|---|---|
| **Age** | 1558 | 0.014 | 0.004 | 3.485 | <.001 | 1.01 | 1.01 | 1.02 |
| **Sex** | | | | | | | | |
| Male | 973 | | | | | | | |
| Female | 585 | -0.549 | 0.163 | -3.375 | 0.001 | 0.58 | 0.42 | 0.79 |
| **CRS auditory scale** | 1558 | 0.26 | 0.109 | 2.374 | 0.018 | 1.3 | 1.05 | 1.61 |
| **CRS visual scale** | 1558 | 0.201 | 0.076 | 2.653 | 0.008 | 1.22 | 1.05 | 1.42 |
| **CRS motor scale** | 1558 | 0.34 | 0.063 | 5.413 | <.001 | 1.4 | 1.24 | 1.59 |
| **CRS communication scale** | 1558 | -0.245 | 0.241 | -1.016 | 0.31 | 0.78 | 0.49 | 1.26 |
| **CRS oromotor/verbal scale** | 1558 | 0.306 | 0.12 | 2.545 | 0.011 | 1.36 | 1.07 | 1.72 |
| CRS arousal scale | 1558 | -0.43 | 0.104 | -4.118 | <.001 | 0.65 | 0.53 | 0.8 |
| **Etiology** | | | | | | | | |
| non-TBI | 951 | | | | | | | |
| TBI | 607 | 0.219 | 0.166 | 1.319 | 0.187 | 1.24 | 0.9 | 1.72 |
| | | | **1825 days** | | | | | |
| **Age** | 1558 | 0.007 | 0.003 | 2.455 | 0.014 | 1.01 | 1 | 1.01 |
| **Sex** | | | | | | | | |
| Male | 973 | | | | | | | |
| Female | 585 | -0.314 | 0.121 | -2.595 | 0.009 | 0.73 | 0.58 | 0.93 |
| **CRS auditory scale** | 1558 | 0.248 | 0.073 | 3.41 | 0.001 | 1.28 | 1.11 | 1.48 |
| **CRS visual scale** | 1558 | 0.249 | 0.052 | 4.818 | <.001 | 1.28 | 1.16 | 1.42 |
| **CRS motor scale** | 1558 | 0.304 | 0.049 | 6.16 | <.001 | 1.35 | 1.23 | 1.49 |
| **CRS communication scale** | 1558 | -0.249 | 0.147 | -1.69 | 0.091 | 0.78 | 0.58 | 1.04 |
| **CRS oromotor/verbal scale** | 1558 | 0.112 | 0.082 | 1.365 | 0.172 | 1.12 | 0.95 | 1.32 |
| CRS arousal scale | 1558 | -0.183 | 0.085 | -2.157 | 0.031 | 0.83 | 0.71 | 0.98 |
| **Etiology** | | | | | | | | |
| non-TBI | 951 | | | | | | | |
| TBI | 607 | 0.198 | 0.094 | 2.106 | 0.035 | 1.22 | 1.01 | 1.47 |
| | | | **3650 days** | | | | | |
| **Age** | 1558 | 0.007 | 0.003 | 2.557 | 0.011 | 1.01 | 1 | 1.01 |
| **Sex** | | | | | | | | |
| Male | 973 | | | | | | | |
| Female | 585 | -0.293 | 0.112 | -2.601 | 0.009 | 0.75 | 0.6 | 0.93 |
| CRS auditory scale | 1558 | 0.241 | 0.076 | 3.164 | 0.002 | 1.27 | 1.1 | 1.48 |
| CRS visual scale | 1558 | 0.253 | 0.049 | 5.161 | <.001 | 1.29 | 1.17 | 1.42 |
| CRS motor scale | 1558 | 0.308 | 0.043 | 7.185 | <.001 | 1.36 | 1.25 | 1.48 |

| | | | | | | | | |
|---|---|---|---|---|---|---|---|---|
| CRS communication scale | 1558 | -0.198 | 0.139 | -1.422 | 0.155 | 0.82 | 0.62 | 1.08 |
| CRS oromotor/verbal scale | 1558 | 0.069 | 0.089 | 0.777 | 0.437 | 1.07 | 0.9 | 1.28 |
| CRS arousal scale | 1558 | -0.226 | 0.082 | -2.743 | 0.006 | 0.8 | 0.68 | 0.94 |
| **Etiology** | | | | | | | | |
| non-TBI | 951 | | | | | | | |
| TBI | 607 | 0.181 | 0.111 | 1.63 | 0.103 | 1.2 | 0.96 | 1.49 |

Table S6 - CRS-R scales, 3 etiologies

| Effect | n | b | SE | z | p | HR | CI lower | CI upper |
|---|---|---|---|---|---|---|---|---|
| | | | | **90 days** | | | | |
| **Age** | 1341 | 0.032 | 0.019 | 1.721 | 0.085 | 1.03 | 1 | 1.07 |
| **Sex** | | | | | | | | |
| Male | 855 | | | | | | | |
| Female | 486 | -0.156 | 0.584 | -0.268 | 0.789 | 0.86 | 0.27 | 2.69 |
| CRS auditory scale | 1341 | 0.105 | 0.318 | 0.329 | 0.742 | 1.11 | 0.6 | 2.07 |
| CRS visual scale | 1341 | 0.249 | 0.195 | 1.28 | 0.2 | 1.28 | 0.88 | 1.88 |
| CRS motor scale | 1341 | -0.095 | 0.235 | -0.406 | 0.685 | 0.91 | 0.57 | 1.44 |
| CRS communication scale | 1341 | -1.948 | 5.605 | -0.348 | 0.728 | 0.14 | 0 | 8420.18 |
| CRS oromotor/verbal scale | 1341 | 0.972 | 0.428 | 2.271 | 0.023 | 2.64 | 1.14 | 6.11 |
| CRS arousal scale | 1341 | -0.568 | 0.339 | -1.676 | 0.094 | 0.57 | 0.29 | 1.1 |
| **Etiology** | | | | | | | | |
| Anoxic | 365 | | | | | | | |
| TBI | 607 | 0.793 | 2.021 | 0.392 | 0.695 | 2.21 | 0.04 | 116.18 |
| Vascular | 369 | 0.749 | 1.944 | 0.385 | 0.7 | 2.11 | 0.05 | 95.38 |
| | | | | **180 days** | | | | |
| **Age** | 1341 | 0.015 | 0.007 | 2.239 | 0.025 | 1.01 | 1 | 1.03 |
| **Sex** | | | | | | | | |
| Male | 855 | | | | | | | |
| Female | 486 | -0.548 | 0.238 | -2.305 | 0.021 | 0.58 | 0.36 | 0.92 |
| CRS auditory scale | 1341 | 0.24 | 0.152 | 1.577 | 0.115 | 1.27 | 0.94 | 1.71 |
| CRS visual scale | 1341 | 0.123 | 0.094 | 1.305 | 0.192 | 1.13 | 0.94 | 1.36 |
| CRS motor scale | 1341 | 0.492 | 0.092 | 5.337 | <.001 | 1.64 | 1.37 | 1.96 |
| CRS communication scale | 1341 | -0.429 | 0.314 | -1.364 | 0.172 | 0.65 | 0.35 | 1.21 |
| CRS oromotor/verbal scale | 1341 | 0.589 | 0.196 | 3.008 | 0.003 | 1.8 | 1.23 | 2.65 |

| | | | | | | | | |
|---|---|---|---|---|---|---|---|---|
| CRS arousal scale | 1341 | -0.473 | 0.181 | -2.616 | 0.009 | 0.62 | 0.44 | 0.89 |
| **Etiology** | | | | | | | | |
| Anoxic | 365 | | | | | | | |
| TBI | 607 | 0.705 | 0.282 | 2.502 | 0.012 | 2.02 | 1.16 | 3.51 |
| Vascular | 369 | 0.456 | 0.333 | 1.37 | 0.171 | 1.58 | 0.82 | 3.03 |
| **365 days** | | | | | | | | |
| **Age** | 1341 | 0.011 | 0.005 | 2.043 | 0.041 | 1.01 | 1 | 1.02 |
| **Sex** | | | | | | | | |
| Male | 855 | | | | | | | |
| Female | 486 | -0.595 | 0.216 | -2.762 | 0.006 | 0.55 | 0.36 | 0.84 |
| **CRS auditory scale** | 1341 | 0.216 | 0.109 | 1.984 | 0.047 | 1.24 | 1 | 1.54 |
| **CRS visual scale** | 1341 | 0.137 | 0.084 | 1.625 | 0.104 | 1.15 | 0.97 | 1.35 |
| **CRS motor scale** | 1341 | 0.426 | 0.065 | 6.597 | <.001 | 1.53 | 1.35 | 1.74 |
| **CRS communication scale** | 1341 | -0.238 | 0.296 | -0.803 | 0.422 | 0.79 | 0.44 | 1.41 |
| **CRS oromotor/verbal scale** | 1341 | 0.437 | 0.155 | 2.823 | 0.005 | 1.55 | 1.14 | 2.1 |
| **CRS arousal scale** | 1341 | -0.358 | 0.158 | -2.263 | 0.024 | 0.7 | 0.51 | 0.95 |
| **Etiology** | | | | | | | | |
| Anoxic | 365 | | | | | | | |
| TBI | 607 | 0.886 | 0.267 | 3.314 | 0.001 | 2.43 | 1.44 | 4.1 |
| Vascular | 369 | 0.875 | 0.322 | 2.72 | 0.007 | 2.4 | 1.28 | 4.51 |
| **1825 days** | | | | | | | | |
| **Age** | 1341 | 0.003 | 0.003 | 1.122 | 0.262 | 1 | 1 | 1.01 |
| **Sex** | | | | | | | | |
| Male | 855 | | | | | | | |
| Female | 486 | -0.264 | 0.107 | -2.47 | 0.014 | 0.77 | 0.62 | 0.95 |
| **CRS auditory scale** | 1341 | 0.255 | 0.078 | 3.26 | 0.001 | 1.29 | 1.11 | 1.51 |
| **CRS visual scale** | 1341 | 0.201 | 0.056 | 3.573 | <.001 | 1.22 | 1.09 | 1.37 |
| **CRS motor scale** | 1341 | 0.344 | 0.047 | 7.26 | <.001 | 1.41 | 1.28 | 1.55 |
| **CRS communication scale** | 1341 | -0.206 | 0.166 | -1.238 | 0.216 | 0.81 | 0.59 | 1.13 |
| **CRS oromotor/verbal scale** | 1341 | 0.13 | 0.092 | 1.422 | 0.155 | 1.14 | 0.95 | 1.36 |
| **CRS arousal scale** | 1341 | -0.098 | 0.104 | -0.938 | 0.348 | 0.91 | 0.74 | 1.11 |
| **Etiology** | | | | | | | | |
| Anoxic | 365 | | | | | | | |
| TBI | 607 | 0.731 | 0.181 | 4.038 | <.001 | 2.08 | 1.46 | 2.96 |
| Vascular | 369 | 0.884 | 0.178 | 4.981 | <.001 | 2.42 | 1.71 | 3.43 |

| | | | | | | | | |
|---|---|---|---|---|---|---|---|---|
| | | | | **3650 days** | | | | |
| **Age** | 1341 | 0.003 | 0.003 | 1.02 | 0.308 | 1 | 1 | 1.01 |
| **Sex** | | | | | | | | |
| Male | 855 | | | | | | | |
| Female | 486 | -0.261 | 0.105 | -2.486 | 0.013 | 0.77 | 0.63 | 0.95 |
| **CRS auditory scale** | 1341 | 0.249 | 0.082 | 3.029 | 0.002 | 1.28 | 1.09 | 1.51 |
| **CRS visual scale** | 1341 | 0.206 | 0.049 | 4.225 | <.001 | 1.23 | 1.12 | 1.35 |
| **CRS motor scale** | 1341 | 0.352 | 0.055 | 6.385 | <.001 | 1.42 | 1.28 | 1.58 |
| **CRS communication scale** | 1341 | -0.165 | 0.174 | -0.952 | 0.341 | 0.85 | 0.6 | 1.19 |
| **CRS oromotor/verbal scale** | 1341 | 0.081 | 0.091 | 0.891 | 0.373 | 1.08 | 0.91 | 1.3 |
| **CRS arousal scale** | 1341 | -0.158 | 0.086 | -1.851 | 0.064 | 0.85 | 0.72 | 1.01 |
| **Etiology** | | | | | | | | |
| Anoxic | 365 | | | | | | | |
| TBI | 607 | 0.703 | 0.175 | 4.018 | <.001 | 2.02 | 1.43 | 2.85 |
| Vascular | 369 | 0.861 | 0.191 | 4.501 | <.001 | 2.37 | 1.63 | 3.44 |

Table S7 - CRS-R index, 2 etiologies

| Effect | n | b | SE | z | p | HR | CI lower | CI upper |
|---|---|---|---|---|---|---|---|---|
| | | | | **90 days** | | | | |
| **Age** | 1558 | 0.033 | 0.011 | 3.171 | 0.002 | 1.03 | 1.01 | 1.06 |
| **Sex** | | | | | | | | |
| Male | 973 | Female | 585 | | | | | |
| Female | 585 | -0.236 | 0.386 | -0.611 | 0.541 | 0.79 | 0.37 | 1.68 |
| **CRS index** | 1558 | 0.025 | 0.008 | 3.333 | 0.001 | 1.03 | 1.01 | 1.04 |
| **Etiology** | | | | | | | | |
| non-TBI | 951 | | | | | | | |
| TBI | 607 | -0.226 | 0.356 | -0.636 | 0.525 | 0.8 | 0.4 | 1.6 |
| | | | | **180 days** | | | | |
| **Age** | 1558 | 0.023 | 0.006 | 4.191 | <.001 | 1.02 | 1.01 | 1.03 |
| **Sex** | | | | | | | | |
| Male | 973 | | | | | | | |
| Female | 585 | -0.402 | 0.19 | -2.113 | 0.035 | 0.67 | 0.46 | 0.97 |
| **CRS index** | 1558 | 0.034 | 0.003 | 11.03 | <.001 | 1.03 | 1.03 | 1.04 |
| **Etiology** | | | | | | | | |

| | | | | | | | | |
|---|---|---|---|---|---|---|---|---|
| non-TBI | 951 | | | | | | | |
| TBI | 607 | 0.338 | 0.169 | 2.004 | 0.045 | 1.4 | 1.01 | 1.95 |
| | | | | **365 days** | | | | |
| **Age** | 1558 | 0.016 | 0.004 | 3.722 | <.001 | 1.02 | 1.01 | 1.02 |
| **Sex** | | | | | | | | |
| Male | 973 | | | | | | | |
| Female | 585 | -0.501 | 0.182 | -2.755 | 0.006 | 0.61 | 0.42 | 0.87 |
| **CRS index** | 1558 | 0.032 | 0.003 | 12.11 | <.001 | 1.03 | 1.03 | 1.04 |
| **Etiology** | | | | | | | | |
| non-TBI | 951 | | | | | | | |
| TBI | 607 | 0.222 | 0.152 | 1.461 | 0.144 | 1.25 | 0.93 | 1.68 |
| | | | | **1825 days** | | | | |
| **Age** | 1558 | 0.007 | 0.003 | 2.854 | 0.004 | 1.01 | 1 | 1.01 |
| **Sex** | | | | | | | | |
| Male | 973 | | | | | | | |
| Female | 585 | -0.268 | 0.109 | -2.452 | 0.014 | 0.76 | 0.62 | 0.95 |
| **CRS index** | 1558 | 0.032 | 0.002 | 17.43 | <.001 | 1.03 | 1.03 | 1.04 |
| **Etiology** | | | | | | | | |
| non-TBI | 951 | | | | | | | |
| TBI | 607 | 0.248 | 0.106 | 2.338 | 0.019 | 1.28 | 1.04 | 1.58 |
| | | | | **3650 days** | | | | |
| **Age** | 1558 | 0.007 | 0.002 | 2.884 | 0.004 | 1.01 | 1 | 1.01 |
| **Sex** | | | | | | | | |
| Male | 973 | | | | | | | |
| Female | 585 | -0.255 | 0.097 | -2.637 | 0.008 | 0.77 | 0.64 | 0.94 |
| **CRS index** | 1558 | 0.032 | 0.002 | 18.15 | <.001 | 1.03 | 1.03 | 1.04 |
| **Etiology** | | | | | | | | |
| non-TBI | 951 | | | | | | | |
| TBI | 607 | 0.234 | 0.096 | 2.436 | 0.015 | 1.26 | 1.05 | 1.53 |

Table S8 - CRS-R index, 3 etiologies

| Effect | n | b | SE | z | p | HR | CI lower | CI upper |
|---|---|---|---|---|---|---|---|---|
| | | | | **90 days** | | | | |
| **Age** | 1341 | 0.036 | 0.017 | 2.11 | 0.035 | 1.04 | 1 | 1.07 |
| **Sex** | | | | | | | | |

| | | | | | | | | |
|---|---|---|---|---|---|---|---|---|
| Male | 855 | Female | 486 | | | | | |
| Female | 486 | -0.071 | 0.538 | -0.133 | 0.894 | 0.93 | 0.32 | 2.67 |
| **CRS index** | 1341 | 0.008 | 0.011 | 0.792 | 0.428 | 1.01 | 0.99 | 1.03 |
| **Etiology** | | | | | | | | |
| Anoxic | 365 | | | | | | | |
| TBI | 607 | 0.845 | 2.26 | 0.374 | 0.709 | 2.33 | 0.03 | 195.35 |
| Vascular | 369 | 0.749 | 2.239 | 0.334 | 0.738 | 2.11 | 0.03 | 170.28 |
| | | | **180 days** | | | | | |
| **Age** | 1341 | 0.019 | 0.006 | 3.368 | 0.001 | 1.02 | 1.01 | 1.03 |
| **Sex** | | | | | | | | |
| Male | 855 | | | | | | | |
| Female | 486 | -0.479 | 0.252 | -1.9 | 0.057 | 0.62 | 0.38 | 1.02 |
| **CRS index** | 1341 | 0.036 | 0.004 | 9.154 | <.001 | 1.04 | 1.03 | 1.04 |
| **Etiology** | | | | | | | | |
| Anoxic | 365 | | | | | | | |
| TBI | 607 | 0.725 | 0.32 | 2.266 | 0.023 | 2.06 | 1.1 | 3.86 |
| Vascular | 369 | 0.404 | 0.331 | 1.223 | 0.221 | 1.5 | 0.78 | 2.87 |
| | | | **365 days** | | | | | |
| **Age** | 1341 | 0.013 | 0.005 | 2.756 | 0.006 | 1.01 | 1 | 1.02 |
| **Sex** | | | | | | | | |
| Male | 855 | | | | | | | |
| Female | 486 | -0.535 | 0.184 | -2.916 | 0.004 | 0.59 | 0.41 | 0.84 |
| **CRS index** | 1341 | 0.033 | 0.003 | 9.643 | <.001 | 1.03 | 1.03 | 1.04 |
| **Etiology** | | | | | | | | |
| Anoxic | 365 | | | | | | | |
| TBI | 607 | 0.924 | 0.269 | 3.437 | 0.001 | 2.52 | 1.49 | 4.27 |
| Vascular | 369 | 0.853 | 0.309 | 2.763 | 0.006 | 2.35 | 1.28 | 4.3 |
| | | | **1825 days** | | | | | |
| **Age** | 1341 | 0.003 | 0.003 | 0.847 | 0.397 | 1 | 1 | 1.01 |
| **Sex** | | | | | | | | |
| Male | 855 | | | | | | | |
| Female | 486 | -0.213 | 0.107 | -1.992 | 0.046 | 0.81 | 0.66 | 1 |
| **CRS index** | 1341 | 0.033 | 0.002 | 15.34 | <.001 | 1.03 | 1.03 | 1.04 |
| **Etiology** | | | | | | | | |
| Anoxic | 365 | | | | | | | |
| TBI | 607 | 0.801 | 0.17 | 4.718 | <.001 | 2.23 | 1.6 | 3.11 |

| | | | | | | | | |
|---|---|---|---|---|---|---|---|---|
| Vascular | 369 | 0.925 | 0.195 | 4.755 | <.001 | 2.52 | 1.72 | 3.69 |
| | | | | **3650 days** | | | | |
| **Age** | 1341 | 0.003 | 0.003 | 0.88 | 0.379 | 1 | 1 | 1.01 |
| **Sex** | | | | | | | | |
| Male | 855 | | | | | | | |
| Female | 486 | -0.213 | 0.108 | -1.976 | 0.048 | 0.81 | 0.65 | 1 |
| **CRS index** | 1341 | 0.032 | 0.002 | 16.34 | <.001 | 1.03 | 1.03 | 1.04 |
| **Etiology** | | | | | | | | |
| Anoxic | 365 | | | | | | | |
| TBI | 607 | 0.774 | 0.154 | 5.041 | <.001 | 2.17 | 1.6 | 2.93 |
| Vascular | 369 | 0.898 | 0.189 | 4.75 | <.001 | 2.46 | 1.69 | 3.56 |

# After cluster correction

Table S9 - Diagnosis, 2 etiologies

| Effect | n | b | SE | z | p | HR | CI lower | CI upper |
|---|---|---|---|---|---|---|---|---|
| | | | | **90 days** | | | | |
| **Age** | 3082 | 0.02 | 0.018 | 1.123 | 0.262 | 1.02 | 0.99 | 1.06 |
| **Sex** | | | | | | | | |
| Male | 2028 | | | | | | | |
| Female | 1054 | -0.248 | 0.211 | -1.175 | 0.24 | 0.78 | 0.52 | 1.18 |
| **Diagnosis** | | | | | | | | |
| MCS | 1260 | | | | | | | |
| UWS | 1822 | -0.8 | 0.439 | -1.823 | 0.068 | 0.45 | 0.19 | 1.06 |
| **Etiology** | | | | | | | | |
| non-TBI | 1667 | | | | | | | |
| TBI | 1415 | 0.182 | 0.322 | 0.564 | 0.573 | 1.2 | 0.64 | 2.26 |
| | | | | **180 days** | | | | |
| **Age** | 3082 | 0.007 | 0.008 | 0.896 | 0.37 | 1.01 | 0.99 | 1.02 |
| **Sex** | | | | | | | | |
| Male | 2028 | | | | | | | |
| Female | 1054 | -0.116 | 0.112 | -1.037 | 0.3 | 0.89 | 0.72 | 1.11 |
| **Diagnosis** | | | | | | | | |
| MCS | 1260 | | | | | | | |
| UWS | 1822 | -0.938 | 0.252 | -3.726 | <.001 | 0.39 | 0.24 | 0.64 |

| | | | | | | | | |
|---|---|---|---|---|---|---|---|---|
| **Etiology** | | | | | | | | |
| non-TBI | 1667 | | | | | | | |
| TBI | 1415 | 0.603 | 0.242 | 2.497 | 0.013 | 1.83 | 1.14 | 2.94 |
| | | | | **365 days** | | | | |
| **Age** | 3082 | 0.005 | 0.006 | 0.787 | 0.431 | 1 | 0.99 | 1.02 |
| **Sex** | | | | | | | | |
| Male | 2028 | | | | | | | |
| Female | 1054 | -0.279 | 0.091 | -3.065 | 0.002 | 0.76 | 0.63 | 0.9 |
| **Diagnosis** | | | | | | | | |
| MCS | 1260 | | | | | | | |
| UWS | 1822 | -1.032 | 0.196 | -5.25 | <.001 | 0.36 | 0.24 | 0.52 |
| **Etiology** | | | | | | | | |
| non-TBI | 1667 | | | | | | | |
| TBI | 1415 | 0.451 | 0.174 | 2.59 | 0.01 | 1.57 | 1.12 | 2.21 |
| | | | | **1825 days** | | | | |
| **Age** | 3082 | 0.003 | 0.004 | 0.617 | 0.537 | 1 | 0.99 | 1.01 |
| **Sex** | | | | | | | | |
| Male | 2028 | | | | | | | |
| Female | 1054 | -0.134 | 0.073 | -1.824 | 0.068 | 0.87 | 0.76 | 1.01 |
| **Diagnosis** | | | | | | | | |
| MCS | 1260 | | | | | | | |
| UWS | 1822 | -1.1 | 0.142 | -7.765 | <.001 | 0.33 | 0.25 | 0.44 |
| **Etiology** | | | | | | | | |
| non-TBI | 1667 | | | | | | | |
| TBI | 1415 | 0.342 | 0.107 | 3.202 | 0.001 | 1.41 | 1.14 | 1.73 |
| | | | | **3650 days** | | | | |
| **Age** | 3082 | 0.002 | 0.004 | 0.565 | 0.572 | 1 | 0.99 | 1.01 |
| **Sex** | | | | | | | | |
| Male | 2028 | | | | | | | |
| Female | 1054 | -0.119 | 0.075 | -1.588 | 0.112 | 0.89 | 0.77 | 1.03 |
| **Diagnosis** | | | | | | | | |
| MCS | 1260 | | | | | | | |
| UWS | 1822 | -1.099 | 0.145 | -7.568 | <.001 | 0.33 | 0.25 | 0.44 |
| **Etiology** | | | | | | | | |
| non-TBI | 1667 | | | | | | | |
| TBI | 1415 | 0.316 | 0.103 | 3.056 | 0.002 | 1.37 | 1.12 | 1.68 |

Table S10 - Diagnosis, 3 etiologies

| Effect | n | b | SE | z | p | HR | CI lower | CI upper |
|---|---|---|---|---|---|---|---|---|
| | | | | **90 days** | | | | |
| **Age** | 2693 | - | - | - | - | - | - | - |
| **Sex** | | | | | | | | |
| Male | 1808 | Female | 885 | | | | | |
| Female | 885 | - | - | - | - | - | - | - |
| **Diagnosis** | | | | | | | | |
| MCS | 1084 | | | | | | | |
| UWS | 1609 | - | - | - | - | - | - | - |
| **Etiology** | | | | | | | | |
| Anoxic | 596 | | | | | | | |
| TBI | 1415 | - | - | - | - | - | - | - |
| Vascular | 682 | - | - | - | - | - | - | - |
| | | | | **180 days** | | | | |
| **Age** | 2693 | 0.002 | 0.008 | 0.27 | 0.787 | 1 | 0.99 | 1.02 |
| **Sex** | | | | | | | | |
| Male | 1808 | | | | | | | |
| Female | 885 | -0.077 | 0.125 | -0.615 | 0.538 | 0.93 | 0.72 | 1.18 |
| **Diagnosis** | | | | | | | | |
| MCS | 1084 | | | | | | | |
| UWS | 1609 | -0.867 | 0.274 | -3.165 | 0.002 | 0.42 | 0.25 | 0.72 |
| **Etiology** | | | | | | | | |
| Anoxic | 596 | | | | | | | |
| TBI | 1415 | 1.215 | 0.384 | 3.165 | 0.002 | 3.37 | 1.59 | 7.15 |
| Vascular | 682 | 0.817 | 0.367 | 2.225 | 0.026 | 2.26 | 1.1 | 4.65 |
| | | | | **365 days** | | | | |
| **Age** | 2693 | 0.001 | 0.006 | 0.115 | 0.908 | 1 | 0.99 | 1.01 |
| **Sex** | | | | | | | | |
| Male | 1808 | | | | | | | |
| Female | 885 | -0.273 | 0.105 | -2.604 | 0.009 | 0.76 | 0.62 | 0.93 |
| **Diagnosis** | | | | | | | | |
| MCS | 1084 | | | | | | | |
| UWS | 1609 | -0.961 | 0.199 | -4.825 | <.001 | 0.38 | 0.26 | 0.57 |
| **Etiology** | | | | | | | | |

| | | | | | | | | |
|---|---|---|---|---|---|---|---|---|
| Anoxic | 596 | | | | | | | |
| TBI | 1415 | 1.078 | 0.31 | 3.482 | <.001 | 2.94 | 1.6 | 5.39 |
| Vascular | 682 | 0.907 | 0.285 | 3.183 | 0.001 | 2.48 | 1.42 | 4.33 |
| | | | | **1825 days** | | | | |
| **Age** | 2693 | -0.001 | 0.004 | -0.353 | 0.724 | 1 | 0.99 | 1.01 |
| **Sex** | | | | | | | | |
| Male | 1808 | | | | | | | |
| Female | 885 | -0.128 | 0.071 | -1.804 | 0.071 | 0.88 | 0.77 | 1.01 |
| **Diagnosis** | | | | | | | | |
| MCS | 1084 | | | | | | | |
| UWS | 1609 | -1.069 | 0.149 | -7.152 | <.001 | 0.34 | 0.26 | 0.46 |
| **Etiology** | | | | | | | | |
| Anoxic | 596 | | | | | | | |
| TBI | 1415 | 0.859 | 0.181 | 4.736 | <.001 | 2.36 | 1.65 | 3.37 |
| Vascular | 682 | 0.825 | 0.157 | 5.269 | <.001 | 2.28 | 1.68 | 3.1 |
| | | | | **3650 days** | | | | |
| **Age** | 2693 | -0.002 | 0.004 | -0.402 | 0.688 | 1 | 0.99 | 1.01 |
| **Sex** | | | | | | | | |
| Male | 1808 | | | | | | | |
| Female | 885 | -0.118 | 0.087 | -1.36 | 0.174 | 0.89 | 0.75 | 1.05 |
| **Diagnosis** | | | | | | | | |
| MCS | 1084 | | | | | | | |
| UWS | 1609 | -1.063 | 0.155 | -6.848 | <.001 | 0.35 | 0.25 | 0.47 |
| **Etiology** | | | | | | | | |
| Anoxic | 596 | | | | | | | |
| TBI | 1415 | 0.819 | 0.2 | 4.097 | <.001 | 2.27 | 1.53 | 3.36 |
| Vascular | 682 | 0.795 | 0.167 | 4.759 | <.001 | 2.21 | 1.6 | 3.07 |

Table S11 – MCS+-, 2 etiologies

| Effect | n | b | SE | z | p | HR | CI lower | CI upper |
|---|---|---|---|---|---|---|---|---|
| | | | | **90 days** | | | | |
| **Age** | 842 | - | - | - | - | - | - | - |
| **Sex** | | | | | | | | |
| Male | 549 | | | | | | | |
| Female | 293 | - | - | - | - | - | - | - |

| | | | | | | | | |
|---|---|---|---|---|---|---|---|---|
| **Diagnosis** | | | | | | | | |
| MCS- | 657 | | | | | | | |
| MCS+ | 185 | - | - | - | - | - | - | - |
| **Etiology** | | | | | | | | |
| non-TBI | 419 | | | | | | | |
| TBI | 423 | - | - | - | - | - | - | - |
| | | | **180 days** | | | | | |
| **Age** | 842 | 0.016 | 0.017 | 0.975 | 0.33 | 1.02 | 0.98 | 1.05 |
| **Sex** | | | | | | | | |
| Male | 549 | | | | | | | |
| Female | 293 | -0.336 | 0.271 | -1.238 | 0.216 | 0.71 | 0.42 | 1.22 |
| **Diagnosis** | | | | | | | | |
| MCS- | 657 | | | | | | | |
| MCS+ | 185 | -0.464 | 0.519 | -0.894 | 0.371 | 0.63 | 0.23 | 1.74 |
| **Etiology** | | | | | | | | |
| non-TBI | 419 | | | | | | | |
| TBI | 423 | 0.249 | 0.283 | 0.881 | 0.378 | 1.28 | 0.74 | 2.23 |
| | | | **365 days** | | | | | |
| **Age** | 842 | 0.012 | 0.012 | 1.041 | 0.298 | 1.01 | 0.99 | 1.04 |
| **Sex** | | | | | | | | |
| Male | 549 | | | | | | | |
| Female | 293 | -0.407 | 0.186 | -2.191 | 0.028 | 0.67 | 0.46 | 0.96 |
| **Diagnosis** | | | | | | | | |
| MCS- | 657 | | | | | | | |
| MCS+ | 185 | 0.158 | 0.54 | 0.293 | 0.769 | 1.17 | 0.41 | 3.38 |
| **Etiology** | | | | | | | | |
| non-TBI | 419 | | | | | | | |
| TBI | 423 | -0.045 | 0.181 | -0.25 | 0.803 | 0.96 | 0.67 | 1.36 |
| | | | **1825 days** | | | | | |
| **Age** | 842 | 0.009 | 0.005 | 1.741 | 0.082 | 1.01 | 1 | 1.02 |
| **Sex** | | | | | | | | |
| Male | 549 | | | | | | | |
| Female | 293 | -0.268 | 0.096 | -2.791 | 0.005 | 0.76 | 0.63 | 0.92 |
| **Diagnosis** | | | | | | | | |
| MCS- | 657 | | | | | | | |
| MCS+ | 185 | 0.019 | 0.256 | 0.073 | 0.942 | 1.02 | 0.62 | 1.68 |

| | | | | | | | | |
|---|---|---|---|---|---|---|---|---|
| **Etiology** | | | | | | | | |
| non-TBI | 419 | | | | | | | |
| TBI | 423 | -0.088 | 0.128 | -0.688 | 0.492 | 0.92 | 0.71 | 1.18 |
| | | | | **3650 days** | | | | |
| **Age** | 842 | 0.009 | 0.006 | 1.638 | 0.102 | 1.01 | 1 | 1.02 |
| **Sex** | | | | | | | | |
| Male | 549 | | | | | | | |
| Female | 293 | -0.259 | 0.107 | -2.425 | 0.015 | 0.77 | 0.63 | 0.95 |
| **Diagnosis** | | | | | | | | |
| MCS- | 657 | | | | | | | |
| MCS+ | 185 | 0.061 | 0.248 | 0.245 | 0.806 | 1.06 | 0.65 | 1.73 |
| **Etiology** | | | | | | | | |
| non-TBI | 419 | | | | | | | |
| TBI | 423 | -0.116 | 0.122 | -0.949 | 0.343 | 0.89 | 0.7 | 1.13 |

Table S12 - CRS-R scales, 2 etiologies

| Effect | n | b | SE | z | p | HR | CI lower | CI upper |
|---|---|---|---|---|---|---|---|---|
| | | | | **90 days** | | | | |
| **Age** | 1558 | - | - | - | - | - | - | - |
| **Sex** | | | | | | | | |
| Male | 973 | | | | | | | |
| Female | 585 | - | - | - | - | - | - | - |
| CRS auditory scale | 1558 | - | - | - | - | - | - | - |
| CRS visual scale | 1558 | - | - | - | - | - | - | - |
| CRS motor scale | 1558 | - | - | - | - | - | - | - |
| CRS communication scale | 1558 | - | - | - | - | - | - | - |
| CRS oromotor/verbal scale | 1558 | - | - | - | - | - | - | - |
| CRS arousal scale | 1558 | - | - | - | - | - | - | - |
| **Etiology** | | | | | | | | |
| non-TBI | 951 | | | | | | | |
| TBI | 607 | - | - | - | - | - | - | - |
| | | | | **180 days** | | | | |
| **Age** | 1558 | - | - | - | - | - | - | - |
| **Sex** | | | | | | | | |
| Male | 973 | | | | | | | |

| | | | | | | | | |
|---|---|---|---|---|---|---|---|---|
| Female | 585 | - | - | - | - | - | - | - |
| CRS auditory scale | 1558 | - | - | - | - | - | - | - |
| CRS visual scale | 1558 | - | - | - | - | - | - | - |
| CRS motor scale | 1558 | - | - | - | - | - | - | - |
| CRS communication scale | 1558 | - | - | - | - | - | - | - |
| CRS oromotor/verbal scale | 1558 | - | - | - | - | - | - | - |
| CRS arousal scale | 1558 | - | - | - | - | - | - | - |
| **Etiology** | | | | | | | | |
| non-TBI | 951 | | | | | | | |
| TBI | 607 | - | - | - | - | - | - | - |
| | | | | **365 days** | | | | |
| **Age** | 1558 | 0.014 | 0.012 | 1.204 | 0.228 | 1.01 | 0.99 | 1.04 |
| **Sex** | | | | | | | | |
| Male | 973 | | | | | | | |
| Female | 585 | -0.549 | 0.206 | -2.673 | 0.008 | 0.58 | 0.39 | 0.86 |
| CRS auditory scale | 1558 | 0.26 | 0.169 | 1.536 | 0.125 | 1.3 | 0.93 | 1.81 |
| CRS visual scale | 1558 | 0.201 | 0.098 | 2.042 | 0.041 | 1.22 | 1.01 | 1.48 |
| CRS motor scale | 1558 | 0.34 | 0.137 | 2.475 | 0.013 | 1.4 | 1.07 | 1.84 |
| CRS communication scale | 1558 | -0.245 | 0.428 | -0.574 | 0.566 | 0.78 | 0.34 | 1.81 |
| CRS oromotor/verbal scale | 1558 | 0.306 | 0.257 | 1.191 | 0.234 | 1.36 | 0.82 | 2.24 |
| CRS arousal scale | 1558 | -0.43 | 0.365 | -1.179 | 0.238 | 0.65 | 0.32 | 1.33 |
| **Etiology** | | | | | | | | |
| non-TBI | 951 | | | | | | | |
| TBI | 607 | 0.219 | 0.194 | 1.129 | 0.259 | 1.24 | 0.85 | 1.82 |
| | | | | **1825 days** | | | | |
| **Age** | 1558 | 0.007 | 0.007 | 1.011 | 0.312 | 1.01 | 0.99 | 1.02 |
| **Sex** | | | | | | | | |
| Male | 973 | | | | | | | |
| Female | 585 | -0.314 | 0.104 | -3.015 | 0.003 | 0.73 | 0.6 | 0.9 |
| CRS auditory scale | 1558 | 0.248 | 0.093 | 2.655 | 0.008 | 1.28 | 1.07 | 1.54 |
| CRS visual scale | 1558 | 0.249 | 0.056 | 4.451 | <.001 | 1.28 | 1.15 | 1.43 |
| CRS motor scale | 1558 | 0.304 | 0.065 | 4.657 | <.001 | 1.35 | 1.19 | 1.54 |
| CRS communication scale | 1558 | -0.249 | 0.251 | -0.992 | 0.321 | 0.78 | 0.48 | 1.27 |
| CRS oromotor/verbal scale | 1558 | 0.112 | 0.166 | 0.679 | 0.497 | 1.12 | 0.81 | 1.55 |

| | | | | | | | | |
|---|---|---|---|---|---|---|---|---|
| CRS arousal scale | 1558 | -0.183 | 0.226 | -0.81 | 0.418 | 0.83 | 0.54 | 1.3 |
| **Etiology** | | | | | | | | |
| non-TBI | 951 | | | | | | | |
| TBI | 607 | 0.198 | 0.11 | 1.807 | 0.071 | 1.22 | 0.98 | 1.51 |
| | | | | **3650 days** | | | | |
| **Age** | 1558 | 0.007 | 0.008 | 0.942 | 0.346 | 1.01 | 0.99 | 1.02 |
| **Sex** | | | | | | | | |
| Male | 973 | | | | | | | |
| Female | 585 | -0.293 | 0.091 | -3.228 | 0.001 | 0.75 | 0.62 | 0.89 |
| CRS auditory scale | 1558 | 0.241 | 0.097 | 2.473 | 0.013 | 1.27 | 1.05 | 1.54 |
| CRS visual scale | 1558 | 0.253 | 0.043 | 5.839 | <.001 | 1.29 | 1.18 | 1.4 |
| CRS motor scale | 1558 | 0.308 | 0.072 | 4.292 | <.001 | 1.36 | 1.18 | 1.57 |
| CRS communication scale | 1558 | -0.198 | 0.238 | -0.831 | 0.406 | 0.82 | 0.51 | 1.31 |
| CRS oromotor/verbal scale | 1558 | 0.069 | 0.166 | 0.417 | 0.677 | 1.07 | 0.77 | 1.48 |
| CRS arousal scale | 1558 | -0.226 | 0.217 | -1.041 | 0.298 | 0.8 | 0.52 | 1.22 |
| **Etiology** | | | | | | | | |
| non-TBI | 951 | | | | | | | |
| TBI | 607 | 0.181 | 0.1 | 1.811 | 0.07 | 1.2 | 0.99 | 1.46 |

Table S13 - CRS-R scales, 3 etiologies

| Effect | n | b | SE | z | p | HR | CI lower | CI upper |
|---|---|---|---|---|---|---|---|---|
| | | | | **90 days** | | | | |
| **Age** | 1341 | - | - | - | - | - | - | - |
| **Sex** | | | | | | | | |
| Male | 855 | | | | | | | |
| Female | 486 | - | - | - | - | - | - | - |
| CRS auditory scale | 1341 | - | - | - | - | - | - | - |
| CRS visual scale | 1341 | - | - | - | - | - | - | - |
| CRS motor scale | 1341 | - | - | - | - | - | - | - |
| CRS communication scale | 1341 | - | - | - | - | - | - | - |
| CRS oromotor/verbal scale | 1341 | - | - | - | - | - | - | - |
| CRS arousal scale | 1341 | - | - | - | - | - | - | - |
| **Etiology** | | | | | | | | |
| Anoxic | 365 | | | | | | | |

| | | | | | | | | |
|---|---|---|---|---|---|---|---|---|
| TBI | 607 | - | - | - | - | - | - | - |
| Vascular | 369 | - | - | - | - | - | - | - |
| **180 days** | | | | | | | | |
| **Age** | 1341 | - | - | - | - | - | - | - |
| **Sex** | | | | | | | | |
| Male | 855 | | | | | | | |
| Female | 486 | - | - | - | - | - | - | - |
| CRS auditory scale | 1341 | - | - | - | - | - | - | - |
| CRS visual scale | 1341 | - | - | - | - | - | - | - |
| CRS motor scale | 1341 | - | - | - | - | - | - | - |
| CRS communication scale | 1341 | - | - | - | - | - | - | - |
| CRS oromotor/verbal scale | 1341 | - | - | - | - | - | - | - |
| CRS arousal scale | 1341 | - | - | - | - | - | - | - |
| **Etiology** | | | | | | | | |
| Anoxic | 365 | | | | | | | |
| TBI | 607 | - | - | - | - | - | - | - |
| Vascular | 369 | - | - | - | - | - | - | - |
| **365 days** | | | | | | | | |
| **Age** | 1341 | 0.011 | 0.013 | 0.817 | 0.414 | 1.01 | 0.99 | 1.04 |
| **Sex** | | | | | | | | |
| Male | 855 | | | | | | | |
| Female | 486 | -0.595 | 0.188 | -3.163 | 0.002 | 0.55 | 0.38 | 0.8 |
| **CRS auditory scale** | 1341 | 0.216 | 0.181 | 1.19 | 0.234 | 1.24 | 0.87 | 1.77 |
| **CRS visual scale** | 1341 | 0.137 | 0.109 | 1.256 | 0.209 | 1.15 | 0.93 | 1.42 |
| **CRS motor scale** | 1341 | 0.426 | 0.132 | 3.23 | 0.001 | 1.53 | 1.18 | 1.98 |
| **CRS communication scale** | 1341 | -0.238 | 0.383 | -0.621 | 0.535 | 0.79 | 0.37 | 1.67 |
| **CRS oromotor/verbal scale** | 1341 | 0.437 | 0.293 | 1.488 | 0.137 | 1.55 | 0.87 | 2.75 |
| **CRS arousal scale** | 1341 | -0.358 | 0.303 | -1.181 | 0.238 | 0.7 | 0.39 | 1.27 |
| **Etiology** | | | | | | | | |
| Anoxic | 365 | | | | | | | |
| TBI | 607 | 0.886 | 0.378 | 2.345 | 0.019 | 2.43 | 1.16 | 5.09 |
| Vascular | 369 | 0.875 | 0.415 | 2.11 | 0.035 | 2.4 | 1.06 | 5.41 |
| **1825 days** | | | | | | | | |
| **Age** | 1341 | 0.003 | 0.008 | 0.451 | 0.652 | 1 | 0.99 | 1.02 |
| **Sex** | | | | | | | | |

| | | | | | | | | |
|---|---|---|---|---|---|---|---|---|
| Male | 855 | | | | | | | |
| Female | 486 | -0.264 | 0.105 | -2.513 | 0.012 | 0.77 | 0.62 | 0.94 |
| **CRS auditory scale** | 1341 | 0.255 | 0.101 | 2.53 | 0.011 | 1.29 | 1.06 | 1.57 |
| **CRS visual scale** | 1341 | 0.201 | 0.052 | 3.845 | <.001 | 1.22 | 1.1 | 1.35 |
| **CRS motor scale** | 1341 | 0.344 | 0.064 | 5.339 | <.001 | 1.41 | 1.24 | 1.6 |
| **CRS communication scale** | 1341 | -0.206 | 0.24 | -0.859 | 0.39 | 0.81 | 0.51 | 1.3 |
| **CRS oromotor/verbal scale** | 1341 | 0.13 | 0.193 | 0.676 | 0.499 | 1.14 | 0.78 | 1.66 |
| **CRS arousal scale** | 1341 | -0.098 | 0.241 | -0.406 | 0.685 | 0.91 | 0.57 | 1.45 |
| **Etiology** | | | | | | | | |
| Anoxic | 365 | | | | | | | |
| TBI | 607 | 0.731 | 0.205 | 3.56 | <.001 | 2.08 | 1.39 | 3.11 |
| Vascular | 369 | 0.884 | 0.208 | 4.238 | <.001 | 2.42 | 1.61 | 3.64 |
| | | | | **3650 days** | | | | |
| **Age** | 1341 | 0.003 | 0.008 | 0.383 | 0.702 | 1 | 0.99 | 1.02 |
| **Sex** | | | | | | | | |
| Male | 855 | | | | | | | |
| Female | 486 | -0.261 | 0.105 | -2.478 | 0.013 | 0.77 | 0.63 | 0.95 |
| **CRS auditory scale** | 1341 | 0.249 | 0.093 | 2.668 | 0.008 | 1.28 | 1.07 | 1.54 |
| **CRS visual scale** | 1341 | 0.206 | 0.053 | 3.885 | <.001 | 1.23 | 1.11 | 1.36 |
| **CRS motor scale** | 1341 | 0.352 | 0.064 | 5.525 | <.001 | 1.42 | 1.26 | 1.61 |
| **CRS communication scale** | 1341 | -0.165 | 0.217 | -0.761 | 0.446 | 0.85 | 0.55 | 1.3 |
| **CRS oromotor/verbal scale** | 1341 | 0.081 | 0.177 | 0.456 | 0.648 | 1.08 | 0.77 | 1.54 |
| **CRS arousal scale** | 1341 | -0.158 | 0.197 | -0.803 | 0.422 | 0.85 | 0.58 | 1.26 |
| **Etiology** | | | | | | | | |
| Anoxic | 365 | | | | | | | |
| TBI | 607 | 0.703 | 0.168 | 4.188 | <.001 | 2.02 | 1.45 | 2.81 |
| Vascular | 369 | 0.861 | 0.211 | 4.083 | <.001 | 2.37 | 1.56 | 3.58 |

Table S14 – CRS-R index, 2 etiologies

| Effect | n | b | SE | z | p | HR | CI lower | CI upper |
|---|---|---|---|---|---|---|---|---|
| | | | | **90 days** | | | | |
| **Age** | 1558 | - | - | - | - | - | - | - |
| **Sex** | | | | | | | | |
| Male | 973 | | | | | | | |

| | | | | | | | | |
|---|---|---|---|---|---|---|---|---|
| Female | 585 | - | - | - | - | - | - | - |
| **CRS index** | 1558 | - | - | - | - | - | - | - |
| **Etiology** | | | | | | | | |
| non-TBI | 951 | | | | | | | |
| TBI | 607 | - | - | - | - | - | - | - |
| | | | | **180 days** | | | | |
| **Age** | 1558 | 0.023 | 0.016 | 1.431 | 0.152 | 1.02 | 0.99 | 1.06 |
| **Sex** | | | | | | | | |
| Male | 973 | | | | | | | |
| Female | 585 | -0.402 | 0.314 | -1.283 | 0.2 | 0.67 | 0.36 | 1.24 |
| **CRS index** | 1558 | 0.034 | 0.009 | 3.76 | <.001 | 1.03 | 1.02 | 1.05 |
| **Etiology** | | | | | | | | |
| non-TBI | 951 | | | | | | | |
| TBI | 607 | 0.338 | 0.254 | 1.33 | 0.184 | 1.4 | 0.85 | 2.31 |
| | | | | **365 days** | | | | |
| **Age** | 1558 | 0.016 | 0.012 | 1.313 | 0.189 | 1.02 | 0.99 | 1.04 |
| **Sex** | | | | | | | | |
| Male | 973 | | | | | | | |
| Female | 585 | -0.501 | 0.197 | -2.545 | 0.011 | 0.61 | 0.41 | 0.89 |
| **CRS index** | 1558 | 0.032 | 0.006 | 5.638 | <.001 | 1.03 | 1.02 | 1.04 |
| **Etiology** | | | | | | | | |
| non-TBI | 951 | | | | | | | |
| TBI | 607 | 0.222 | 0.186 | 1.191 | 0.234 | 1.25 | 0.87 | 1.8 |
| | | | | **1825 days** | | | | |
| **Age** | 1558 | 0.007 | 0.008 | 0.952 | 0.341 | 1.01 | 0.99 | 1.02 |
| **Sex** | | | | | | | | |
| Male | 973 | | | | | | | |
| Female | 585 | -0.268 | 0.12 | -2.239 | 0.025 | 0.76 | 0.6 | 0.97 |
| **CRS index** | 1558 | 0.032 | 0.003 | 10.59 | <.001 | 1.03 | 1.03 | 1.04 |
| **Etiology** | | | | | | | | |
| non-TBI | 951 | | | | | | | |
| TBI | 607 | 0.248 | 0.126 | 1.973 | 0.049 | 1.28 | 1 | 1.64 |
| | | | | **3650 days** | | | | |
| **Age** | 1558 | 0.007 | 0.007 | 0.992 | 0.321 | 1.01 | 0.99 | 1.02 |
| **Sex** | | | | | | | | |
| Male | 973 | | | | | | | |

| | | | | | | | | |
|---|---|---|---|---|---|---|---|---|
| Female | 585 | -0.255 | 0.105 | -2.438 | 0.015 | 0.77 | 0.63 | 0.95 |
| **CRS index** | 1558 | 0.032 | 0.004 | 8.939 | <.001 | 1.03 | 1.03 | 1.04 |
| **Etiology** | | | | | | | | |
| non-TBI | 951 | | | | | | | |
| TBI | 607 | 0.234 | 0.103 | 2.262 | 0.024 | 1.26 | 1.03 | 1.55 |

Table S15 – CRS-R index, 3 etiologies

| Effect | n | b | SE | z | p | HR | CI lower | CI upper |
|---|---|---|---|---|---|---|---|---|
| | | | | **90 days** | | | | |
| **Age** | 1341 | - | - | - | - | - | - | - |
| **Sex** | | | | | | | | |
| Male | 855 | - | - | - | - | - | - | - |
| Female | 486 | - | - | - | - | - | - | - |
| **CRS index** | 1341 | - | - | - | - | - | - | - |
| **Etiology** | | | | | | | | |
| Anoxic | 365 | - | - | - | - | - | - | - |
| TBI | 607 | - | - | - | - | - | - | - |
| Vascular | 369 | - | - | - | - | - | - | - |
| | | | | **180 days** | | | | |
| **Age** | 1341 | - | - | - | - | - | - | - |
| **Sex** | | | | | | | | |
| Male | 855 | - | - | - | - | - | - | - |
| Female | 486 | - | - | - | - | - | - | - |
| **CRS index** | 1341 | - | - | - | - | - | - | - |
| **Etiology** | | | | | | | | |
| Anoxic | 365 | - | - | - | - | - | - | - |
| TBI | 607 | - | - | - | - | - | - | - |
| Vascular | 369 | - | - | - | - | - | - | - |
| | | | | **365 days** | | | | |
| **Age** | 1341 | 0.013 | 0.015 | 0.896 | 0.37 | 1.01 | 0.98 | 1.04 |
| **Sex** | | | | | | | | |
| Male | 855 | | | | | | | |
| Female | 486 | -0.535 | 0.22 | -2.437 | 0.015 | 0.59 | 0.38 | 0.9 |
| **CRS index** | 1341 | 0.033 | 0.006 | 5.56 | <.001 | 1.03 | 1.02 | 1.05 |
| **Etiology** | | | | | | | | |

| | | | | | | | | |
|---|---|---|---|---|---|---|---|---|
| Anoxic | 365 | | | | | | | |
| TBI | 607 | 0.924 | 0.539 | 1.712 | 0.087 | 2.52 | 0.87 | 7.25 |
| Vascular | 369 | 0.853 | 0.616 | 1.385 | 0.166 | 2.35 | 0.7 | 7.85 |
| | | | | **1825 days** | | | | |
| **Age** | 1341 | 0.003 | 0.008 | 0.393 | 0.695 | 1 | 0.99 | 1.02 |
| **Sex** | | | | | | | | |
| Male | 855 | | | | | | | |
| Female | 486 | -0.213 | 0.108 | -1.97 | 0.049 | 0.81 | 0.65 | 1 |
| **CRS index** | 1341 | 0.033 | 0.003 | 10.41 | <.001 | 1.03 | 1.03 | 1.04 |
| **Etiology** | | | | | | | | |
| Anoxic | 365 | | | | | | | |
| TBI | 607 | 0.801 | 0.193 | 4.151 | <.001 | 2.23 | 1.53 | 3.25 |
| Vascular | 369 | 0.925 | 0.232 | 3.984 | <.001 | 2.52 | 1.6 | 3.98 |
| | | | | **3650 days** | | | | |
| **Age** | 1341 | 0.003 | 0.009 | 0.318 | 0.751 | 1 | 0.99 | 1.02 |
| **Sex** | | | | | | | | |
| Male | 855 | | | | | | | |
| Female | 486 | -0.213 | 0.123 | -1.731 | 0.083 | 0.81 | 0.63 | 1.03 |
| **CRS index** | 1341 | 0.032 | 0.003 | 10.17 | <.001 | 1.03 | 1.03 | 1.04 |
| **Etiology** | | | | | | | | |
| Anoxic | 365 | | | | | | | |
| TBI | 607 | 0.774 | 0.186 | 4.167 | <.001 | 2.17 | 1.51 | 3.12 |
| Vascular | 369 | 0.898 | 0.241 | 3.732 | <.001 | 2.46 | 1.53 | 3.94 |

# Univariable models

Table S16 - Single predictor models for each time period and overall sample

| Effect | n | b | SE | z | p | HR | CI lower | CI upper |
|---|---|---|---|---|---|---|---|---|
| | | | | **90 days** | | | | |
| **Age** | 3252 | 0.019 | 0.006 | 3.469 | 0.001 | 1.02 | 1.01 | 1.03 |
| **Sex** | | | | | | | | |
| Male | 2029 | | | | | | | |
| Female | 1056 | -0.213 | 0.23 | -0.926 | 0.354 | 0.81 | 0.51 | 1.27 |
| **Diagnosis** | | | | | | | | |

| | | | | | | | | |
|---|---|---|---|---|---|---|---|---|
| MCS | 1312 | | | | | | | |
| UWS | 1978 | -0.755 | 0.207 | -3.654 | <.001 | 0.47 | 0.31 | 0.7 |
| **Diagnosis** | | | | | | | | |
| MCS- | 672 | | | | | | | |
| MCS+ | 205 | -0.468 | 1.005 | -0.465 | 0.642 | 0.63 | 0.09 | 4.49 |
| **Etiology** | | | | | | | | |
| Anoxic | 635 | | | | | | | |
| TBI | 1523 | 1.191 | 0.807 | 1.477 | 0.14 | 3.29 | 0.68 | 16 |
| Vascular | 718 | 1.533 | 0.819 | 1.871 | 0.061 | 4.63 | 0.93 | 23.07 |
| **Etiology** | | | | | | | | |
| non-TBI | 1766 | | | | | | | |
| TBI | 1523 | 0.07 | 0.226 | 0.309 | 0.757 | 1.07 | 0.69 | 1.67 |
| **CRS auditory** | 1582 | 0.634 | 0.139 | 4.568 | <.001 | 1.89 | 1.44 | 2.47 |
| **CRS visual** | 1580 | 0.427 | 0.1 | 4.276 | <.001 | 1.53 | 1.26 | 1.86 |
| **CRS motor** | 1583 | 0.294 | 0.128 | 2.303 | 0.021 | 1.34 | 1.04 | 1.72 |
| **CRS communication** | 1581 | 0.39 | 0.575 | 0.677 | 0.498 | 1.48 | 0.48 | 4.56 |
| **CRS oromotor/verbal** | 1581 | 0.92 | 0.234 | 3.93 | <.001 | 2.51 | 1.59 | 3.97 |
| **CRS arousal** | 1582 | -0.285 | 0.208 | -1.373 | 0.17 | 0.75 | 0.5 | 1.13 |
| **CRS index** | 1579 | 0.026 | 0.006 | 4.027 | <.001 | 1.03 | 1.01 | 1.04 |
| | | | | **180 days** | | | | |
| **Age** | 3252 | 0.002 | 0.003 | 0.885 | 0.376 | 1 | 1 | 1.01 |
| **Sex** | | | | | | | | |
| Male | 2029 | | | | | | | |
| Female | 1056 | -0.208 | 0.121 | -1.715 | 0.086 | 0.81 | 0.64 | 1.03 |
| **Diagnosis** | | | | | | | | |
| MCS | 1312 | | | | | | | |
| UWS | 1978 | -0.953 | 0.095 | -9.982 | <.001 | 0.39 | 0.32 | 0.46 |
| **Diagnosis** | | | | | | | | |
| MCS- | 672 | | | | | | | |
| MCS+ | 205 | -0.656 | 0.273 | -2.401 | 0.016 | 0.52 | 0.3 | 0.89 |
| **Etiology** | | | | | | | | |
| Anoxic | 635 | | | | | | | |
| TBI | 1523 | 1.41 | 0.224 | 6.289 | <.001 | 4.1 | 2.64 | 6.35 |
| Vascular | 718 | 1.058 | 0.242 | 4.369 | <.001 | 2.88 | 1.79 | 4.63 |
| **Etiology** | | | | | | | | |

| | | | | | | | | |
|---|---|---|---|---|---|---|---|---|
| non-TBI | 1766 | | | | | | | |
| TBI | 1523 | 0.618 | 0.102 | 6.061 | <.001 | 1.85 | 1.52 | 2.26 |
| **CRS auditory** | 1582 | 0.636 | 0.079 | 8.029 | <.001 | 1.89 | 1.62 | 2.21 |
| **CRS visual** | 1580 | 0.414 | 0.06 | 6.84 | <.001 | 1.51 | 1.34 | 1.7 |
| **CRS motor** | 1583 | 0.589 | 0.063 | 9.365 | <.001 | 1.8 | 1.59 | 2.04 |
| **CRS communication** | 1581 | 0.913 | 0.196 | 4.653 | <.001 | 2.49 | 1.7 | 3.66 |
| **CRS oromotor/verbal** | 1581 | 0.967 | 0.115 | 8.401 | <.001 | 2.63 | 2.1 | 3.29 |
| **CRS arousal** | 1582 | -0.171 | 0.116 | -1.479 | 0.139 | 0.84 | 0.67 | 1.06 |
| **CRS index** | 1579 | 0.034 | 0.003 | 12.73 | <.001 | 1.03 | 1.03 | 1.04 |
| | | | **365 days** | | | | | |
| **Age** | 3252 | 0 | 0.002 | -0.056 | 0.956 | 1 | 1 | 1 |
| **Sex** | | | | | | | | |
| Male | 2029 | | | | | | | |
| Female | 1056 | -0.358 | 0.092 | -3.87 | <.001 | 0.7 | 0.58 | 0.84 |
| **Diagnosis** | | | | | | | | |
| MCS | 1312 | | | | | | | |
| UWS | 1978 | -0.985 | 0.073 | -13.51 | <.001 | 0.37 | 0.32 | 0.43 |
| **Diagnosis** | | | | | | | | |
| MCS- | 672 | | | | | | | |
| MCS+ | 205 | -0.003 | 0.177 | -0.014 | 0.989 | 1 | 0.7 | 1.41 |
| **Etiology** | | | | | | | | |
| Anoxic | 635 | | | | | | | |
| TBI | 1523 | 1.302 | 0.136 | 9.571 | <.001 | 3.68 | 2.82 | 4.8 |
| Vascular | 718 | 1.085 | 0.152 | 7.112 | <.001 | 2.96 | 2.19 | 3.99 |
| **Etiology** | | | | | | | | |
| non-TBI | 1766 | | | | | | | |
| TBI | 1523 | 0.541 | 0.074 | 7.334 | <.001 | 1.72 | 1.49 | 1.99 |
| **CRS auditory** | 1582 | 0.594 | 0.057 | 10.48 | <.001 | 1.81 | 1.62 | 2.02 |
| **CRS visual** | 1580 | 0.412 | 0.048 | 8.572 | <.001 | 1.51 | 1.37 | 1.66 |
| **CRS motor** | 1583 | 0.536 | 0.053 | 10.11 | <.001 | 1.71 | 1.54 | 1.9 |
| **CRS communication** | 1581 | 0.923 | 0.178 | 5.194 | <.001 | 2.52 | 1.78 | 3.56 |
| **CRS oromotor/verbal** | 1581 | 0.778 | 0.122 | 6.393 | <.001 | 2.18 | 1.72 | 2.76 |
| **CRS arousal** | 1582 | -0.039 | 0.118 | -0.331 | 0.741 | 0.96 | 0.76 | 1.21 |
| **CRS index** | 1579 | 0.032 | 0.003 | 12.1 | <.001 | 1.03 | 1.03 | 1.04 |
| | | | **1825 days** | | | | | |

| | | | | | | | | |
|---|---|---|---|---|---|---|---|---|
| **Age** | 3252 | -0.002 | 0.002 | -1.139 | 0.255 | 1 | 0.99 | 1 |
| **Sex** | | | | | | | | |
| Male | 2029 | | | | | | | |
| Female | 1056 | -0.212 | 0.058 | -3.62 | <.001 | 0.81 | 0.72 | 0.91 |
| **Diagnosis** | | | | | | | | |
| MCS | 1312 | | | | | | | |
| UWS | 1978 | -1.057 | 0.063 | -16.66 | <.001 | 0.35 | 0.31 | 0.39 |
| **Diagnosis** | | | | | | | | |
| MCS- | 672 | | | | | | | |
| MCS+ | 205 | -0.078 | 0.1 | -0.781 | 0.435 | 0.92 | 0.76 | 1.13 |
| **Etiology** | | | | | | | | |
| Anoxic | 635 | | | | | | | |
| TBI | 1523 | 1.189 | 0.115 | 10.35 | <.001 | 3.29 | 2.62 | 4.11 |
| Vascular | 718 | 1.01 | 0.129 | 7.821 | <.001 | 2.74 | 2.13 | 3.53 |
| **Etiology** | | | | | | | | |
| non-TBI | 1766 | | | | | | | |
| TBI | 1523 | 0.506 | 0.063 | 8.097 | <.001 | 1.66 | 1.47 | 1.88 |
| **CRS auditory** | 1582 | 0.581 | 0.051 | 11.33 | <.001 | 1.79 | 1.62 | 1.98 |
| **CRS visual** | 1580 | 0.43 | 0.03 | 14.16 | <.001 | 1.54 | 1.45 | 1.63 |
| **CRS motor** | 1583 | 0.5 | 0.036 | 14.04 | <.001 | 1.65 | 1.54 | 1.77 |
| **CRS communication** | 1581 | 0.867 | 0.108 | 8.006 | <.001 | 2.38 | 1.93 | 2.94 |
| **CRS oromotor/verbal** | 1581 | 0.582 | 0.071 | 8.198 | <.001 | 1.79 | 1.56 | 2.06 |
| **CRS arousal** | 1582 | 0.222 | 0.079 | 2.806 | 0.005 | 1.25 | 1.07 | 1.46 |
| **CRS index** | 1579 | 0.032 | 0.002 | 19.19 | <.001 | 1.03 | 1.03 | 1.04 |
| | | | | **3650 days** | | | | |
| **Age** | 3252 | -0.002 | 0.002 | -1.093 | 0.274 | 1 | 0.99 | 1 |
| **Sex** | | | | | | | | |
| Male | 2029 | | | | | | | |
| Female | 1056 | -0.185 | 0.06 | -3.06 | 0.002 | 0.83 | 0.74 | 0.94 |
| **Diagnosis** | | | | | | | | |
| MCS | 1312 | | | | | | | |
| UWS | 1978 | -1.056 | 0.068 | -15.57 | <.001 | 0.35 | 0.3 | 0.4 |
| **Diagnosis** | | | | | | | | |
| MCS- | 672 | | | | | | | |
| MCS+ | 205 | -0.03 | 0.109 | -0.275 | 0.783 | 0.97 | 0.78 | 1.2 |

| | | | | | | | | |
|---|---|---|---|---|---|---|---|---|
| **Etiology** | | | | | | | | |
| Anoxic | 635 | | | | | | | |
| TBI | 1523 | 1.153 | 0.106 | 10.93 | <.001 | 3.17 | 2.58 | 3.9 |
| Vascular | 718 | 0.98 | 0.122 | 8.01 | <.001 | 2.66 | 2.1 | 3.39 |
| **Etiology** | | | | | | | | |
| non-TBI | 1766 | | | | | | | |
| TBI | 1523 | 0.483 | 0.056 | 8.611 | <.001 | 1.62 | 1.45 | 1.81 |
| **CRS auditory** | 1582 | 0.563 | 0.045 | 12.46 | <.001 | 1.75 | 1.61 | 1.92 |
| **CRS visual** | 1580 | 0.426 | 0.029 | 14.7 | <.001 | 1.53 | 1.45 | 1.62 |
| **CRS motor** | 1583 | 0.49 | 0.043 | 11.48 | <.001 | 1.63 | 1.5 | 1.78 |
| **CRS communication** | 1581 | 0.877 | 0.106 | 8.304 | <.001 | 2.4 | 1.95 | 2.96 |
| **CRS oromotor/verbal** | 1581 | 0.536 | 0.073 | 7.302 | <.001 | 1.71 | 1.48 | 1.97 |
| **CRS arousal** | 1582 | 0.145 | 0.084 | 1.716 | 0.086 | 1.16 | 0.98 | 1.36 |
| **CRS index** | 1579 | 0.032 | 0.002 | 18.87 | <.001 | 1.03 | 1.03 | 1.04 |
| | | | **No time restriction** | | | | | |
| **Age** | 3252 | -0.002 | 0.002 | -1.254 | 0.21 | 1 | 0.99 | 1 |
| **Sex** | | | | | | | | |
| Male | 2029 | | | | | | | |
| Female | 1056 | -0.18 | 0.06 | -3.01 | 0.003 | 0.83 | 0.74 | 0.94 |
| **Diagnosis** | | | | | | | | |
| MCS | 1312 | | | | | | | |
| UWS | 1978 | -1.056 | 0.058 | -18.06 | <.001 | 0.35 | 0.31 | 0.39 |
| **Diagnosis** | | | | | | | | |
| MCS- | 672 | | | | | | | |
| MCS+ | 205 | -0.016 | 0.086 | -0.182 | 0.856 | 0.98 | 0.83 | 1.17 |
| **Etiology** | | | | | | | | |
| Anoxic | 635 | | | | | | | |
| TBI | 1523 | 1.152 | 0.106 | 10.81 | <.001 | 3.16 | 2.57 | 3.9 |
| Vascular | 718 | 0.978 | 0.107 | 9.119 | <.001 | 2.66 | 2.16 | 3.28 |
| **Etiology** | | | | | | | | |
| non-TBI | 1766 | | | | | | | |
| TBI | 1523 | 0.486 | 0.061 | 7.978 | <.001 | 1.63 | 1.44 | 1.83 |
| **CRS auditory** | 1582 | 0.564 | 0.047 | 12.01 | <.001 | 1.76 | 1.6 | 1.93 |
| **CRS visual** | 1580 | 0.422 | 0.032 | 13.25 | <.001 | 1.52 | 1.43 | 1.62 |
| **CRS motor** | 1583 | 0.487 | 0.037 | 13.11 | <.001 | 1.63 | 1.51 | 1.75 |

| | | | | | | | | |
|---|---|---|---|---|---|---|---|---|
| **CRS communication** | 1581 | 0.882 | 0.1 | 8.788 | <.001 | 2.42 | 1.98 | 2.94 |
| **CRS oromotor/verbal** | 1581 | 0.528 | 0.084 | 6.322 | <.001 | 1.7 | 1.44 | 2 |
| **CRS arousal** | 1582 | 0.131 | 0.084 | 1.554 | 0.12 | 1.14 | 0.97 | 1.34 |
| **CRS index** | 1579 | 0.032 | 0.002 | 16.18 | <.001 | 1.03 | 1.03 | 1.04 |

Table S17 – Single predictor models for each time period and overall sample after correction for correlational structure

| Effect | n | b | SE | z | p | HR | CI lower | CI upper |
|---|---|---|---|---|---|---|---|---|
| | | | | **90 days** | | | | |
| **Age** | 3252 | 0.019 | 0.019 | 1.025 | 0.305 | 1.02 | 0.98 | 1.06 |
| **Sex** | | | | | | | | |
| Male | 2029 | | | | | | | |
| Female | 1056 | -0.213 | 0.184 | -1.16 | 0.246 | 0.81 | 0.56 | 1.16 |
| **Diagnosis** | | | | | | | | |
| MCS | 1312 | | | | | | | |
| UWS | 1978 | -0.755 | 0.406 | -1.862 | 0.063 | 0.47 | 0.21 | 1.04 |
| **Diagnosis** | | | | | | | | |
| MCS- | 672 | | | | | | | |
| MCS+ | 205 | - | - | - | - | - | - | - |
| **Etiology** | | | | | | | | |
| Anoxic | 635 | | | | | | | |
| TBI | 1523 | - | - | - | - | - | - | - |
| Vascular | 718 | - | - | - | - | - | - | - |
| **Etiology** | | | | | | | | |
| non-TBI | 1766 | | | | | | | |
| TBI | 1523 | 0.07 | 0.331 | 0.211 | 0.833 | 1.07 | 0.56 | 2.05 |
| **CRS auditory** | 1582 | 0.634 | 0.478 | 1.325 | 0.185 | 1.89 | 0.74 | 4.81 |
| **CRS visual** | 1580 | 0.427 | 0.2 | 2.134 | 0.033 | 1.53 | 1.04 | 2.27 |
| **CRS motor** | 1583 | 0.294 | 0.705 | 0.417 | 0.677 | 1.34 | 0.34 | 5.35 |
| **CRS communication** | 1581 | - | - | - | - | - | - | - |
| **CRS oromotor/verbal** | 1581 | 0.92 | 0.654 | 1.407 | 0.16 | 2.51 | 0.7 | 9.05 |
| **CRS arousal** | 1582 | -0.285 | 0.736 | -0.388 | 0.698 | 0.75 | 0.18 | 3.18 |
| **CRS index** | 1579 | 0.026 | 0.019 | 1.401 | 0.161 | 1.03 | 0.99 | 1.06 |
| | | | | **180 days** | | | | |

| | | | | | | | | |
|---|---|---|---|---|---|---|---|---|
| **Age** | 3252 | 0.002 | 0.009 | 0.271 | 0.786 | 1 | 0.98 | 1.02 |
| **Sex** | | | | | | | | |
| Male | 2029 | | | | | | | |
| Female | 1056 | -0.208 | 0.14 | -1.481 | 0.138 | 0.81 | 0.62 | 1.07 |
| **Diagnosis** | | | | | | | | |
| MCS | 1312 | | | | | | | |
| UWS | 1978 | -0.953 | 0.235 | -4.05 | <.001 | 0.39 | 0.24 | 0.61 |
| **Diagnosis** | | | | | | | | |
| MCS- | 672 | | | | | | | |
| MCS+ | 205 | -0.656 | 0.559 | -1.174 | 0.24 | 0.52 | 0.17 | 1.55 |
| **Etiology** | | | | | | | | |
| Anoxic | 635 | | | | | | | |
| TBI | 1523 | 1.41 | 0.316 | 4.459 | <.001 | 4.1 | 2.2 | 7.61 |
| Vascular | 718 | 1.058 | 0.276 | 3.837 | <.001 | 2.88 | 1.68 | 4.94 |
| **Etiology** | | | | | | | | |
| non-TBI | 1766 | | | | | | | |
| TBI | 1523 | 0.618 | 0.249 | 2.48 | 0.013 | 1.85 | 1.14 | 3.02 |
| **CRS auditory** | 1582 | 0.636 | 0.143 | 4.454 | <.001 | 1.89 | 1.43 | 2.5 |
| **CRS visual** | 1580 | 0.414 | 0.091 | 4.533 | <.001 | 1.51 | 1.26 | 1.81 |
| **CRS motor** | 1583 | 0.589 | 0.169 | 3.488 | <.001 | 1.8 | 1.29 | 2.51 |
| **CRS communication** | 1581 | 0.913 | 0.428 | 2.135 | 0.033 | 2.49 | 1.08 | 5.76 |
| **CRS oromotor/verbal** | 1581 | 0.967 | 0.486 | 1.987 | 0.047 | 2.63 | 1.01 | 6.82 |
| **CRS arousal** | 1582 | -0.171 | 0.658 | -0.26 | 0.795 | 0.84 | 0.23 | 3.06 |
| **CRS index** | 1579 | 0.034 | 0.008 | 4.435 | <.001 | 1.03 | 1.02 | 1.05 |
| | | | | **365 days** | | | | |
| **Age** | 3252 | 0 | 0.006 | -0.018 | 0.986 | 1 | 0.99 | 1.01 |
| **Sex** | | | | | | | | |
| Male | 2029 | | | | | | | |
| Female | 1056 | -0.358 | 0.086 | -4.177 | <.001 | 0.7 | 0.59 | 0.83 |
| **Diagnosis** | | | | | | | | |
| MCS | 1312 | | | | | | | |
| UWS | 1978 | -0.985 | 0.183 | -5.372 | <.001 | 0.37 | 0.26 | 0.54 |
| **Diagnosis** | | | | | | | | |
| MCS- | 672 | | | | | | | |
| MCS+ | 205 | -0.003 | 0.552 | -0.005 | 0.996 | 1 | 0.34 | 2.94 |

| | | | | | | | | |
|---|---|---|---|---|---|---|---|---|
| **Etiology** | | | | | | | | |
| Anoxic | 635 | | | | | | | |
| TBI | 1523 | 1.302 | 0.241 | 5.396 | <.001 | 3.68 | 2.29 | 5.9 |
| Vascular | 718 | 1.085 | 0.217 | 4.992 | <.001 | 2.96 | 1.93 | 4.53 |
| **Etiology** | | | | | | | | |
| non-TBI | 1766 | | | | | | | |
| TBI | 1523 | 0.541 | 0.152 | 3.558 | <.001 | 1.72 | 1.28 | 2.31 |
| **CRS auditory** | 1582 | 0.594 | 0.13 | 4.584 | <.001 | 1.81 | 1.4 | 2.33 |
| **CRS visual** | 1580 | 0.412 | 0.071 | 5.77 | <.001 | 1.51 | 1.31 | 1.74 |
| **CRS motor** | 1583 | 0.536 | 0.103 | 5.198 | <.001 | 1.71 | 1.4 | 2.09 |
| **CRS communication** | 1581 | 0.923 | 0.309 | 2.986 | 0.003 | 2.52 | 1.37 | 4.61 |
| **CRS oromotor/verbal** | 1581 | 0.778 | 0.289 | 2.691 | 0.007 | 2.18 | 1.24 | 3.84 |
| **CRS arousal** | 1582 | -0.039 | 0.321 | -0.122 | 0.903 | 0.96 | 0.51 | 1.81 |
| **CRS index** | 1579 | 0.032 | 0.005 | 6.153 | <.001 | 1.03 | 1.02 | 1.04 |
| | | | | **1825 days** | | | | |
| **Age** | 3252 | -0.002 | 0.004 | -0.492 | 0.622 | 1 | 0.99 | 1.01 |
| **Sex** | | | | | | | | |
| Male | 2029 | | | | | | | |
| Female | 1056 | -0.212 | 0.072 | -2.948 | 0.003 | 0.81 | 0.7 | 0.93 |
| **Diagnosis** | | | | | | | | |
| MCS | 1312 | | | | | | | |
| UWS | 1978 | -1.057 | 0.149 | -7.075 | <.001 | 0.35 | 0.26 | 0.47 |
| **Diagnosis** | | | | | | | | |
| MCS- | 672 | | | | | | | |
| MCS+ | 205 | -0.078 | 0.244 | -0.321 | 0.748 | 0.92 | 0.57 | 1.49 |
| **Etiology** | | | | | | | | |
| Anoxic | 635 | | | | | | | |
| TBI | 1523 | 1.189 | 0.151 | 7.882 | <.001 | 3.29 | 2.44 | 4.42 |
| Vascular | 718 | 1.01 | 0.14 | 7.203 | <.001 | 2.74 | 2.09 | 3.61 |
| **Etiology** | | | | | | | | |
| non-TBI | 1766 | | | | | | | |
| TBI | 1523 | 0.506 | 0.1 | 5.053 | <.001 | 1.66 | 1.36 | 2.02 |
| **CRS auditory** | 1582 | 0.581 | 0.076 | 7.663 | <.001 | 1.79 | 1.54 | 2.07 |
| **CRS visual** | 1580 | 0.43 | 0.045 | 9.606 | <.001 | 1.54 | 1.41 | 1.68 |
| **CRS motor** | 1583 | 0.5 | 0.06 | 8.263 | <.001 | 1.65 | 1.46 | 1.86 |

| | | | | | | | | |
|---|---|---|---|---|---|---|---|---|
| **CRS communication** | 1581 | 0.867 | 0.276 | 3.137 | 0.002 | 2.38 | 1.38 | 4.09 |
| **CRS oromotor/verbal** | 1581 | 0.582 | 0.194 | 2.998 | 0.003 | 1.79 | 1.22 | 2.62 |
| **CRS arousal** | 1582 | 0.222 | 0.229 | 0.97 | 0.332 | 1.25 | 0.8 | 1.95 |
| **CRS index** | 1579 | 0.032 | 0.004 | 9.008 | <.001 | 1.03 | 1.03 | 1.04 |
| | | | | **3650 days** | | | | |
| **Age** | 3252 | -0.002 | 0.004 | -0.487 | 0.626 | 1 | 0.99 | 1.01 |
| **Sex** | | | | | | | | |
| Male | 2029 | | | | | | | |
| Female | 1056 | -0.185 | 0.079 | -2.357 | 0.018 | 0.83 | 0.71 | 0.97 |
| **Diagnosis** | | | | | | | | |
| MCS | 1312 | | | | | | | |
| UWS | 1978 | -1.056 | 0.141 | -7.474 | <.001 | 0.35 | 0.26 | 0.46 |
| **Diagnosis** | | | | | | | | |
| MCS- | 672 | | | | | | | |
| MCS+ | 205 | -0.03 | 0.258 | -0.116 | 0.908 | 0.97 | 0.58 | 1.61 |
| **Etiology** | | | | | | | | |
| Anoxic | 635 | | | | | | | |
| TBI | 1523 | 1.153 | 0.162 | 7.114 | <.001 | 3.17 | 2.31 | 4.35 |
| Vascular | 718 | 0.98 | 0.155 | 6.328 | <.001 | 2.66 | 1.97 | 3.61 |
| **Etiology** | | | | | | | | |
| non-TBI | 1766 | | | | | | | |
| TBI | 1523 | 0.483 | 0.104 | 4.649 | <.001 | 1.62 | 1.32 | 1.99 |
| **CRS auditory** | 1582 | 0.563 | 0.097 | 5.83 | <.001 | 1.75 | 1.45 | 2.12 |
| **CRS visual** | 1580 | 0.426 | 0.053 | 8.066 | <.001 | 1.53 | 1.38 | 1.7 |
| **CRS motor** | 1583 | 0.49 | 0.059 | 8.312 | <.001 | 1.63 | 1.45 | 1.83 |
| **CRS communication** | 1581 | 0.877 | 0.299 | 2.932 | 0.003 | 2.4 | 1.34 | 4.32 |
| **CRS oromotor/verbal** | 1581 | 0.536 | 0.155 | 3.465 | 0.001 | 1.71 | 1.26 | 2.31 |
| **CRS arousal** | 1582 | 0.145 | 0.164 | 0.88 | 0.379 | 1.16 | 0.84 | 1.59 |
| **CRS index** | 1579 | 0.032 | 0.003 | 9.395 | <.001 | 1.03 | 1.03 | 1.04 |
| | | | | **No time restriction** | | | | |
| **Age** | 3252 | -0.002 | 0.004 | -0.503 | 0.615 | 1 | 0.99 | 1.01 |
| **Sex** | | | | | | | | |
| Male | 2029 | | | | | | | |
| Female | 1056 | -0.18 | 0.072 | -2.502 | 0.012 | 0.83 | 0.72 | 0.96 |
| **Diagnosis** | | | | | | | | |

| | | | | | | | | |
|---|---|---|---|---|---|---|---|---|
| MCS | 1312 | | | | | | | |
| UWS | 1978 | -1.056 | 0.127 | -8.334 | <.001 | 0.35 | 0.27 | 0.45 |
| **Diagnosis** | | | | | | | | |
| MCS- | 672 | | | | | | | |
| MCS+ | 205 | -0.016 | 0.22 | -0.071 | 0.943 | 0.98 | 0.64 | 1.52 |
| **Etiology** | | | | | | | | |
| Anoxic | 635 | | | | | | | |
| TBI | 1523 | 1.152 | 0.154 | 7.473 | <.001 | 3.16 | 2.34 | 4.28 |
| Vascular | 718 | 0.978 | 0.163 | 5.992 | <.001 | 2.66 | 1.93 | 3.66 |
| **Etiology** | | | | | | | | |
| non-TBI | 1766 | | | | | | | |
| TBI | 1523 | 0.486 | 0.098 | 4.949 | <.001 | 1.63 | 1.34 | 1.97 |
| **CRS auditory** | 1582 | 0.564 | 0.092 | 6.141 | <.001 | 1.76 | 1.47 | 2.1 |
| **CRS visual** | 1580 | 0.422 | 0.05 | 8.381 | <.001 | 1.52 | 1.38 | 1.68 |
| **CRS motor** | 1583 | 0.487 | 0.059 | 8.222 | <.001 | 1.63 | 1.45 | 1.83 |
| **CRS communication** | 1581 | 0.882 | 0.285 | 3.095 | 0.002 | 2.42 | 1.38 | 4.22 |
| **CRS oromotor/verbal** | 1581 | 0.528 | 0.184 | 2.873 | 0.004 | 1.7 | 1.18 | 2.43 |
| **CRS arousal** | 1582 | 0.131 | 0.155 | 0.847 | 0.397 | 1.14 | 0.84 | 1.54 |
| **CRS index** | 1579 | 0.032 | 0.003 | 9.902 | <.001 | 1.03 | 1.03 | 1.04 |

Notes. - indicates non convergence of the model and lack of reliable information about the coefficients, p-values, and CI.

Table S18 – Corrected and uncorrected for correlational structure univariable and multivariable models of age for 3650 time period separated by diagnosis (UWS vs MCS)

| Effect | n | b | SE | z | p | HR | CI lower | CI upper |
|---|---|---|---|---|---|---|---|---|
| | | | | **UWS, multivariable** | | | | |
| **Age** | 1822 | -0.01 | 0.003 | -2.992 | 0.003 | 0.99 | 0.98 | 1 |
| **Etiology** | | | | | | | | |
| non-TBI | 1081 | | | | | | | |
| TBI | 741 | 0.756 | 0.102 | 7.388 | <.001 | 2.13 | 1.74 | 2.6 |
| **Sex** | | | | | | | | |
| Male | 1179 | | | | | | | |
| Female | 643 | -0.034 | 0.122 | -0.282 | 0.778 | 0.97 | 0.76 | 1.23 |
| | | | | **UWS, multivariable, cluster corrected** | | | | |
| **Age** | 1822 | -0.01 | 0.004 | -2.372 | 0.018 | 0.99 | 0.98 | 1 |
| **Etiology** | | | | | | | | |
| non-TBI | 1081 | | | | | | | |
| TBI | 741 | 0.756 | 0.156 | 4.861 | <.001 | 2.13 | 1.57 | 2.89 |
| **Sex** | | | | | | | | |
| Male | 1179 | | | | | | | |
| Female | 643 | -0.034 | 0.103 | -0.334 | 0.739 | 0.97 | 0.79 | 1.18 |
| | | | | **MCS, multivariable** | | | | |
| **Age** | 1260 | 0.005 | 0.002 | 2.014 | 0.044 | 1 | 1 | 1.01 |
| **Etiology** | | | | | | | | |
| non-TBI | 586 | | | | | | | |
| TBI | 674 | 0.029 | 0.065 | 0.452 | 0.651 | 1.03 | 0.91 | 1.17 |
| **Sex** | | | | | | | | |
| Male | 849 | | | | | | | |
| Female | 411 | -0.156 | 0.089 | -1.748 | 0.081 | 0.86 | 0.72 | 1.02 |
| | | | | **MCS, multivariable, cluster corrected** | | | | |
| **Age** | 1260 | 0.005 | 0.004 | 1.188 | 0.235 | 1 | 1 | 1.01 |
| **Etiology** | | | | | | | | |
| non-TBI | 586 | | | | | | | |

| Effect | n | b | SE | z | p | HR | CI lower | CI upper |
|---|---|---|---|---|---|---|---|---|
| TBI | 674 | 0.029 | 0.107 | 0.274 | 0.784 | 1.03 | 0.84 | 1.27 |
| **Sex** | | | | | | | | |
| Male | 849 | | | | | | | |
| Female | 411 | -0.156 | 0.07 | -2.24 | 0.025 | 0.86 | 0.75 | 0.98 |
| | | | | **UWS, univariable** | | | | |
| **Age** | 1941 | -0.016 | 0.003 | -5.066 | <.001 | 0.98 | 0.98 | 0.99 |
| | | | | **UWS, univariable, cluster corrected** | | | | |
| **Age** | 1941 | -0.016 | 0.005 | -3.374 | <.001 | 0.98 | 0.98 | 0.99 |
| | | | | **MCS, univariable** | | | | |
| **Age** | 1311 | 0.004 | 0.002 | 2.047 | 0.041 | 1.00 | 1.00 | 1.01 |
| | | | | **MCS, univariable, cluster corrected** | | | | |
| **Age** | 1311 | 0.004 | 0.005 | 0.803 | 0.422 | 1.00 | 0.99 | 1.01 |

Table S19 – Univariable model of CRS-R auditory subscale treated as a categorical variable with two different reference levels

| Effect | n | b | SE | z | p | HR | CI lower | CI upper |
|---|---|---|---|---|---|---|---|---|
| | | | | **3650 days** | | | | |
| | | | | **CRS-R auditory** | | | | |
| Reference: 0 | 246 | | | | | | | |
| 1 | 768 | -0.113 | 0.165 | -0.687 | 0.492 | 0.89 | 0.63 | 1.27 |
| 2 | 317 | 0.673 | 0.179 | 3.753 | <0.001 | 1.96 | 1.40 | 2.74 |
| 3 | 210 | 1.51 | 0.173 | 8.727 | <0.001 | 4.53 | 3.17 | 6.47 |
| 4 | 41 | 1.318 | 0.258 | 5.108 | <0.001 | 3.73 | 2.24 | 6.22 |
| Reference: 2 | 317 | | | | | | | |
| 0 | 246 | -0.673 | 0.189 | -3.564 | <0.001 | 0.51 | 0.35 | 0.74 |
| 1 | 768 | -0.786 | 0.126 | -6.235 | <0.001 | 0.46 | 0.36 | 0.58 |
| 3 | 210 | 0.837 | 0.125 | 6.706 | <0.001 | 2.31 | 1.81 | 2.95 |
| 4 | 41 | 0.645 | 0.185 | 3.484 | <0.001 | 1.91 | 1.36 | 2.74 |
